# Home-administered unfocused transcranial ultrasound in subjective cognitive decline and dementia: an open-label, uncontrolled feasibility pilot study

**DOI:** 10.3389/fnagi.2026.1937897

**Published:** 2026-09-28

**Authors:** Helané Wahbeh, Sterling Cooley, Steven Russell Chamberlin, Thomas Brophy, Stuart Hameroff

**Affiliations:** 1 Research Department, Institute of Noetic Sciences, Novato, CA, United States; 2 Department of Neurology, Oregon Health and Science University, Portland, OR, United States; 3 Ultra DAO, Berkeley, CA, United States; 4 Quantum Life Technologies Inc., San Francisco, CA, United States

**Keywords:** Alzheimer’s disease, cognitive impairment, dementia, memory, microtubules, neuromodulation, pilot study, transcranial ultrasound

## Abstract

**Background:**

Memory and cognitive impairments remain disabling, with few effective treatments. Low-intensity transcranial ultrasound (TUS) is a non-invasive neuromodulation approach with a favorable safety profile and preliminary evidence of cognitive benefit, with one hypothesized but unproven mechanism involving microtubule resonance and reassembly.

**Objectives:**

This open-label pilot study evaluated the safety and preliminary efficacy of the US Pro2000 unfocused ultrasound device in adults with memory or cognitive difficulties, a sample spanning subjective cognitive decline among self-administering participants to diagnosed dementia among caregiver-rated recipients, and described self-reported symptom trajectories.

**Methods:**

In a within-participant design, adults with self-reported cognitive impairment, or their caregivers, applied the device at the right temporal window for 5 min three times daily (target 15 min/day), in cycles of 3 weeks on and 1 week off. Outcomes (GAD-7, CESD-5, Inoue battery, and caregiver-rated MDS 3.0) and adverse events were collected online. Mixed-effects models with age and device-use covariates assessed change over time, with False Discovery Rate correction within aim.

**Results:**

Twenty-seven participants enrolled and 26 initiated treatment. Across 2,174 daily assessments, no serious or unanticipated adverse events occurred; only headache (15.4%) and transient jaw clenching (3.8%) were possibly related, and none caused discontinuation. Among self-administered participants, depression (CESD-5) declined significantly (*p*_FDR = 0.016) and five of eight monthly self-report symptoms improved, including memory, word-finding, and problem-solving (all *p*_FDR ≤ 0.040); anxiety trended downward, and the objective cognitive battery, near ceiling at baseline, did not change. Caregiver-rated outcomes were stable. Device use showed no dose response, and sensitivity analyses were consistent.

**Conclusion:**

Home-based self-administered low-intensity TUS was well tolerated, with only mild transient events, although adverse-event ascertainment relied on unblinded self-report and relatedness judgments were made by unblinded investigators. Efficacy signals were confined to self-report, while objective cognition and caregiver ratings were unchanged. The uncontrolled design, small subgroups, and reliance on self-report preclude causal inference. These findings support the safety and feasibility of TUS and justify an adequately powered, double-blind, sham-controlled trial.

## 1 Introduction

Alzheimer’s disease and related dementias impose a substantial burden on healthcare systems and on affected individuals and their families (Sontheimer et al., 2021). Despite decades of research, pharmacological strategies targeting extracellular beta-amyloid plaques have produced limited therapeutic success (Walsh and Selkoe, 2020). Attention has increasingly turned to intracellular pathology, particularly neurofibrillary tangles of hyperphosphorylated tau, which correlate more strongly with the severity of cognitive impairment. Tau is a microtubule-associated protein that helps stabilize microtubules and supports their role in synaptic plasticity, which is essential for memory and cognition (Dixit et al., 2008).

Microtubules are self-assembling cylindrical polymers of the protein tubulin, the brain’s most abundant protein (tied with actin), and are found in all animal and plant cells. They provide structural support, establish and regulate synapses, and are theorized to encode memory (Craddock et al., 2012) and support consciousness (Hameroff and Penrose, 2014; Hameroff, 2022). These memory-encoding and consciousness proposals are contested and are considered as hypothesis in section “4.9 Hypothesized Mechanism: Microtubule Resonance.” Free tau and neurofibrillary tangles are signs of microtubule disassembly, which may produce the synapse loss and cognitive deficits seen in Alzheimer’s disease. This hypothesis is long-standing, articulated by Matsuyama and Jarvik (1989), but overshadowed by decades of amyloid-focused work. Efforts to stabilize microtubules pharmacologically have shown promise in cellular and animal models (Ballatore et al., 2012; Barten et al., 2012; Brunden et al., 2012; Zhang et al., 2012, 2018; Fernandez-Valenzuela et al., 2020), but clinical translation has been difficult: agents such as epothilone D and TPI-287 have been limited by tolerability problems and halted trials (Tsai et al., 2020), motivating non-pharmacological approaches to restoring microtubule function.

Microtubules also resonate over a wide range of frequencies, and *in vitro* the rate of tubulin assembly into microtubules peaks under electromagnetic fields in the megahertz range (Sahu et al., 2014). Because electromagnetic fields penetrate the skull poorly, ultrasound offers a way to deliver mechanical oscillation in that same range noninvasively. This rationale motivated the present study although it remains hypothetical and untested in the intact human brain.

### 1.1 Transcranial ultrasound

Ultrasound, mechanical vibration above 20 kilohertz, penetrates the skull far more effectively than electromagnetic fields at comparable frequencies and can therefore be delivered noninvasively to the brain. Ultrasound applied to the brain through the scalp and skull (‘transcranial ultrasound’, TUS) is strongly attenuated in a frequency-dependent manner, with transmission falling as frequency rises; at the 1 MHz operating frequency of the US Pro2000, only a fraction of the incident acoustic energy reaches the cortex, although the temporal window targeted here is among the most acoustically favorable regions of the skull (White et al., 2006). Even so, the transmitted energy is sufficient to produce measurable physiological and behavioral effects (Tyler et al., 2008; Sarica et al., 2022).

TUS can be focused or unfocused and delivered at high, medium, or low intensity. High-intensity focused ultrasound can heat tissue to create lesions, for example, to ablate tumors; medium intensity can open the blood-brain barrier; and low, sub-thermal intensity, as used in the present study, modulates brain function without heating. Ultrasound has been used safely in medical imaging for decades, including through the infant fontanelle (Vitale et al., 2020).

Low-intensity ultrasound has promoted axonal regeneration and memory-related dendritic spine formation in animal models (Wen et al., 2021; Winkler-Ferenczi et al., 2024). It can also excite neurons by activating membrane mechanosensitive ion channels (Yoo et al., 2022) and has been shown to enhance clearance of interstitial and cerebrospinal fluid from the brain (Yoo et al., 2023).

The first human double-blind study of TUS effects on mental states, conducted by one of the present authors (SH) and colleagues in patients with chronic pain, found that 15 s of unfocused 8 MHz TUS to the temporal scalp produced mood enhancement and pain reduction lasting more than 40 min (Hameroff et al., 2013). Subsequent work has shown that TUS can modulate cortical regions and deep structures, including the thalamus and brainstem, with focused stimulation of the right prefrontal cortex improving mood and altering functional connectivity (Sanguinetti et al., 2020).

### 1.2 Preliminary evidence in cognitive impairment

Preliminary studies suggest TUS may benefit cognitive impairment. In animal models of Alzheimer’s disease, medium intensity ultrasound improved cognition and reduced pathology (Leinenga and Götz, 2015). In 35 patients with Alzheimer’s disease, another study reported gains in memory and verbal function using transcranial pulse stimulation, an ultrashort-pulse modality that is mechanistically distinct from the continuous unfocused ultrasound used here (Beisteiner et al., 2020). A follow-up analysis in the same cohort reported increased cortical thickness alongside clinical improvement, though the uncontrolled design leaves the direction of that association unresolved (Popescu et al., 2021). In four patients with Alzheimer’s disease, low-intensity hippocampal-directed focused TUS was associated with increased cortical glucose metabolism and mild improvements in memory, executive, and global cognitive function (Jeong et al., 2021). Whether unfocused TUS can match focused or pulse-based approaches for cognitive enhancement is unknown, as no comparative data exist; its low cost and accessibility nonetheless motivate systematic evaluation. It should also be noted that none of the human ultrasound and cognition studies cited in this section (section “1.2 Preliminary Evidence in Cognitive Impairment”) was sham-controlled, so this literature shares the expectancy vulnerabilities that constrain the present study.

### 1.3 Safety of TUS

A systematic review by Sarica et al. (2022) examined 35 TUS studies (2013 to 2022) involving 677 participants across healthy adults, chronic pain, dementia, epilepsy, traumatic brain injury, and depression. No persistent or serious adverse events were reported. Transient mild events occurred in 3.4% (14 of 425) of those systematically surveyed, including headache, mood change, scalp heating, cognitive symptoms, neck pain, muscle twitches, anxiety, sleepiness, and pruritus, all resolving without lasting consequence. Additional reviews corroborate the safety of low-intensity ultrasound, and FDA diagnostic-ultrasound limits, including a spatial-peak pulse-average intensity ceiling of 190 W/cm^2^, can be translated to therapeutic TUS (Miller et al., 2012; Radjenovic et al., 2022).

### 1.4 Present study

This open-label pilot study evaluates the safety and preliminary efficacy of the US Pro2000 unfocused ultrasound device, self-administered transcranially at the right temporal window, in adults with memory and cognitive difficulties. Data were collected from individuals treating themselves and from caregivers treating a care recipient. The study was designed to inform the feasibility, safety profile, and preliminary effect estimates needed to plan a future double-blind randomized controlled trial. Three aims were specified in the IRB-approved protocol before enrollment began. The study was not prospectively registered in a public trial registry (section “2.1 Study Design”), and all efficacy analyses should therefore be regarded as exploratory.

Aim 1, Safety: to evaluate the safety profile of the US Pro2000 by monitoring and reporting adverse events. We hypothesized that adverse events would be mild and transient, consistent with the TUS literature, and that no unanticipated serious events would occur.
Aim 2, Efficacy: to evaluate preliminary effects on memory and cognitive symptoms, indexed by validated measures of anxiety (GAD-7), depression (CESD-5), cognition (Inoue Computerized Test Battery), and caregiver-rated cognitive patterns and mood (MDS 3.0, Sections C and D). We hypothesized improvement over time.
Aim 3, Subjective Cognitive Symptom Trajectories: to document the course of self-reported cognitive and quality-of-life symptoms across treatment. We hypothesized significant improvement over time.

## 2 Materials and methods

### 2.1 Study design

This was an exploratory, open-label, within-participant pilot study evaluating the effects of self-administered unfocused transcranial ultrasound in adults experiencing memory or cognitive difficulties. All study activities were approved by the Institute of Noetic Sciences Institutional Review Board (IORG#0003743), and all participants (or their legally authorized representatives) provided informed consent prior to enrollment. Enrollment was rolling, and intervention duration was not fixed; participants continued in the study until they elected to discontinue, ceased device use, or reached the planned 6-month follow-up window. No randomization or blinding was employed. This study was not prospectively registered in a public trial registry such as ClinicalTrials.gov. It was designed and conducted as an open-label feasibility and safety pilot rather than as a hypothesis-testing efficacy trial. The three aims, their hypotheses, and the primary analytic approach were specified in the IRB-approved protocol before enrollment began and are described throughout as protocol-specified rather than pre-registered; the analysis sets and the rate-based sensitivity analysis were defined before analysis but do not appear in that protocol. All efficacy analyses should be regarded as exploratory.

### 2.2 Participants

Participants were recruited through the investigators’ existing networks, as well as through dissemination by the Institute of Noetic Sciences and partner networks via social media and member communications. The study originally targeted enrollment of up to 100 participants. No demographic exclusions were applied beyond the eligibility criteria below.

Because the intervention was self-administered, eligibility was determined by self-report during a screening process and was not confirmed by the participant’s physician beyond a required letter of physician approval.

#### 2.2.1 Inclusion criteria

Eligible participants were: (1) adults aged 18 years or older; (2) English-speaking; (3) experiencing memory impairment, cognitive difficulty, or diagnosed Alzheimer’s disease or related dementia; (4) with daily home access to a US Pro2000 device; (5) already using or planning to use it transcranially for these symptoms; (6) able to provide informed consent, or with a legal representative to consent on their behalf; and (7) able to provide a physician letter of approval. For individuals unable to consent, a primary caregiver consented, administered the intervention and surveys, and documented legal authority (guardianship, durable power of attorney for healthcare, or healthcare proxy). Enrolling both self-administering participants and caregiver-rated recipients allowed feasibility and safety to be assessed across the full range of impairment that a future trial would encounter.

#### 2.2.2 Exclusion criteria

Participants were excluded for: history of traumatic brain injury; diagnosis of schizophrenia or other significant psychiatric disorders; concurrent use of other investigational treatments for memory or cognitive difficulties; severe auditory or visual impairments precluding survey completion; current substance abuse; moderate to severe tinnitus; presence of metal implants or electronic medical devices that could be affected by ultrasound; and history of seizures or bleeding conditions (including current use of blood thinners).

### 2.3 Intervention

Participants self-administered the US Pro2000 device transcranially at the right temporal window, targeting frontotemporal brain regions. The application site is located anterior and superior to the tragus and superior to the zygomatic arch, corresponding to the squamous portion of the temporal bone where the skull is thinnest.

The prescribed protocol was three 5-min sessions per day (15 min total per day). To accommodate participant preferences, an alternative schedule of two 7.5-min sessions per day (totaling 15 min) was permitted upon request, preserving the daily target dose. Treatment was delivered in cycles of 3 consecutive weeks of daily use followed by a 1-week break, and cycles were repeated for the duration of participation. This protocol was developed by one of the investigators (S. Cooley) based on prior anecdotal use; to our knowledge, this study represents the first formal evaluation of this protocol. No parameter of this protocol has independent empirical or mechanistic justification in the published literature. The choice of the right temporal window, the 5-min session duration, the three-times-daily frequency, and the three-weeks-on and one-week-off schedule all derive from the investigator’s informal experience with the device rather than from prior controlled data. The protocol optimization, including laterality, session duration, duty cycle, and on-off scheduling, remains an open empirical question for future dose-finding studies.

The device was operated at the LOW setting throughout. Per manufacturer labeling (Model DU3035), this delivers a collimated, pulsed 1.0 MHz beam with an effective radiating area of 4.0 cm^2^, a spatial-average temporal-average intensity of 0.08 W/cm^2^ ± 20% (0.064 to 0.096 W/cm^2^), a 5% modulation duty cycle at a pulse repetition rate of 100 Hz, giving a pulse duration of 0.5 ms and a spatial-average pulse-average intensity of approximately 1.6 W/cm^2^. With a maximum beam nonuniformity ratio of 5.0, the corresponding spatial-peak values at the transducer face are approximately 0.4 W/cm^2^ temporal-average and 8 W/cm^2^ pulse-average, and the mechanical index is approximately 0.5. These values are derived from manufacturer labeling; acoustic output was not independently calibrated, and neither within-session output stability nor intracranial intensity was measured. The US Pro2000 is regulated as a therapeutic physiotherapy ultrasound unit and was used off-label in this study for transcranial application.

### 2.4 Measures

All measures were administered via a HIPAA-compliant SurveyMonkey platform, accessible by laptop, tablet, or smartphone.

#### 2.4.1 Safety (aim 1)

Adverse events were captured continuously throughout the study through (1) the Daily Assessment Form, which included an item for reporting any anticipated or unanticipated effects; (2) spontaneous self-reports via a dedicated study phone number and email address; (3) caregiver observations; and (4) records maintained by research staff. Each event was characterized by its nature, duration, severity, and resolution.

#### 2.4.2 Efficacy (aim 2) - self-administered outcomes

##### 2.4.2.1 Cognition

The Inoue Computerized Test Battery for Alzheimer’s Disease Screening in Community-based Settings (Inoue et al., 2009) was administered at baseline and monthly. The battery includes a three-word memory test, temporal orientation, three-dimensional visual-spatial perception, and delayed recall, and takes approximately 5–10 min to complete. This battery was selected because the fully remote, unsupervised design required a brief, validated instrument that could be delivered online without an administrator. It was developed as a community screening tool, however, and its restricted score range limits sensitivity to change in mildly impaired samples (see sections “3.4.1 Self-Administered Outcomes” and “4.8 Strengths and Limitations”).

##### 2.4.2.2 Anxiety

The Generalized Anxiety Disorder–7 (GAD-7; Spitzer et al., 2006) was administered at baseline and monthly. The seven items are rated 0 (not at all) to 3 (nearly every day) over the previous 2 weeks; total scores range 0–21, with higher scores indicating greater anxiety.

##### 2.4.2.3 Depression

The 5-item Center for Epidemiologic Studies Depression Scale (CESD-5; Kohout et al., 1993) was administered at baseline and monthly. Items assess depressive symptom frequency over the previous week on a 0–3 scale; total scores are summed across items and multiplied by 4 to match CESD-20 scores, with scores ranging from 0 to 60 and higher scores indicating greater depression severity.

#### 2.4.3 Efficacy (aim 2) - caregiver-administered outcomes

MDS 3.0 Sections C and D. For caregiver-rated participants, the Long-Term Care Facility Resident Assessment Instrument 3.0 (MDS 3.0; Centers for Medicare and Medicaid Services, 2024) was used. Section C (Cognitive Patterns, items C07 to C10) assessed short-term memory, long-term memory, memory/recall ability, and cognitive skills for daily decision-making. Section D (Resident Mood, PHQ-9) comprises nine mood symptoms rated over the prior 2 weeks, with total severity scores from 0 to 27, completed here by the caregiver about the recipient. This measure is referred to as Section D mood (PHQ-9) throughout.

#### 2.4.4 Subjective cognitive symptom trajectories (aim 3)

##### 2.4.4.1 Monthly self-report symptoms

A 7-item self-report symptom assessment was administered at baseline and monthly thereafter, covering memory loss, word-finding difficulty, confusion, problem-solving/planning difficulty, mood or behavior changes, impaired judgment, and social withdrawal. Each item used a continuous visual analog slider (0–100) anchored at Never–Sometimes–Always.

##### 2.4.4.2 Daily assessment form

A daily email link prompted participants to record device-use compliance for the preceding day (whether the device was used; number of sessions [once, twice, three times]; session duration [5 min vs. more than 5 min]) and any positive or negative symptom changes. The form also included a field for reporting adverse events.

### 2.5 Compliance operationalization

Total device-use time was computed from the Daily Assessment Form. Sessions per day were coded 0 to 3, and session duration was 5 or 7.5 min (a reported session longer than 5 min was treated as the authorized 7.5-min alternative). Daily minutes were sessions multiplied by minutes per session, summed across days to yield a total-minutes covariate per participant. Days without a completed Daily Assessment Form were treated as zero minutes for the primary analysis. Compliance was ascertained solely through participant self-report. The device provides no usage-logging capability, so session occurrence, duration, placement, and intensity setting could not be objectively verified.

### 2.6 Analysis sets

Three analysis sets were defined prior to analysis section (“2.1 Study Design”). Safety set (Aim 1): all enrolled participants with evidence of device use or any post-baseline adverse-event information from any source (daily assessments, follow-up surveys, spontaneous reports, caregiver observations, or staff records).

Efficacy set (Aim 2): enrolled participants with baseline data, evidence of device exposure, and at least one post-baseline outcome assessment for the relevant measure.

Subjective Cognitive Symptom trajectories set (Aim 3): for the monthly self-report symptom analyses, participants with baseline plus at least one post-baseline monthly symptom assessment; for daily symptom analyses, participants with baseline plus at least one completed Daily Assessment Form.

Participants who completed screening only were included in recruitment and eligibility counts. Participants who completed baseline but provided no post-baseline data were retained for baseline descriptive reporting and the participant flow diagram but were excluded from efficacy and longitudinal symptom analyses.

### 2.7 Statistical analysis

Analyses were conducted in Stata v15.0 (StataCorp, College Station, TX) and R version 4.3.3; figures were produced in Python (Matplotlib). Descriptive statistics (means, standard deviations, frequencies) summarized demographic characteristics, baseline scores, adverse events, and compliance variables. Normality of each outcome was assessed with the Shapiro–Wilk test.

No formal *a priori* power calculation was performed. The enrollment target of 100 participants was a feasibility and recruitment target for this open-label pilot rather than a power-based estimate, and all efficacy analyses are considered exploratory. Consistent with the aims of a pilot study, effect estimates and their confidence intervals are reported to characterize the magnitude and precision of within-participant change and to inform sample-size planning for an adequately powered, sham-controlled trial rather than to provide confirmatory tests of efficacy.

Baseline characteristics were compared between the self- and caregiver-administered groups: continuous variables (age, years of education, self-rated socioeconomic status) with Wilcoxon rank-sum test; sex with the Fisher’s exact test; and race, collected as a sparse multiple-response item, with the Fisher’s exact test after collapsing to a person-level binary (identification with at least one non-European category vs. European only). These comparisons were exploratory, describing sample composition rather than testing an aim, and were not corrected for multiple comparisons.

In a *post-ho*c, exploratory analysis prompted by the divergent trajectories between the two groups, baseline values of the seven monthly self-report symptom items and their composite index were compared between the self- and caregiver-administered groups using two-sided Wilcoxon rank-sum tests. Because this comparison was added after the primary analyses and was descriptive rather than confirmatory, it was not corrected for multiple comparisons.

For Aim 1, adverse events were summarized as counts and percentages by type.

For Aim 2 and the monthly component of Aim 3, change in each outcome over time was modeled with linear mixed-effects models with random participant intercepts and Satterthwaite denominator degrees of freedom. Fixed effects were time (visit), age, and total device-use minutes (a compliance covariate); age and minutes were mean-centered. These models use all available observations under a missing-at-random assumption and accommodate unequal numbers of observations per participant, features suited to the rolling-enrollment design. As in Aim 2, the Aim 3 monthly symptom outcomes (seven items and their composite) were analyzed separately for the self- and caregiver-administered groups, reflecting the distinction between self-report and caregiver proxy-report and the groups’ differing baseline symptom levels.

Although several outcomes departed from normality on the Shapiro–Wilk test, the mixed-effects models were retained as primary, and GEE (exchangeable working correlation, robust standard errors) were fitted as a sensitivity analysis for the Aim 2 efficacy outcomes; this refines the analysis plan specified in the IRB protocol, in which GEE had been specified as a substitute for the mixed model under non-normality. In the caregiver-administered subgroup (*n* = 8), the binary (C07, C08) and ordinal (C09, C10) MDS outcomes were fitted with linear mixed-effects models rather than logistic or ordinal models, which do not reliably converge at this sample size, so their estimates are approximate; and because GEE robust standard errors are unreliable at this cluster count, the mixed-effects models are treated as primary throughout this subgroup.

Multiplicity was controlled with the Benjamini–Hochberg false discovery rate procedure (Benjamini and Hochberg, 1995), applied to the time (visit) effects within each aim and, for Aims 2 and 3, separately within the self- and caregiver-administered analyses; age and compliance covariate *p*-values are reported unadjusted.

As a sensitivity analysis, the Aim 2 models were re-fitted using mean minutes per study day rather than total minutes to assess sensitivity to the dose–duration relationship inherent in the rolling-enrollment design (section “2.5 Compliance Operationalization”).

For the daily component of Aim 3, the frequency and percentage of positive and negative symptom reports were summarized. Free-text responses to the positive- and negative-change items on the Daily Assessment Form were analyzed thematically: a large language model (Claude Opus 4.8; Anthropic, San Francisco, CA, USA; accessed June 2026) performed an initial inductive pass to surface recurring themes. The model’s role was limited to proposing this candidate coding frame. Every model-generated theme and every comment-to-theme assignment was then independently reviewed by a human coder (HW) against the verbatim source comments, with misclassifications corrected, overlapping categories consolidated, and all representative quotes selected by the human coder directly from the source text; no theme or assignment was retained without human confirmation. Known biases of model-assisted coding, including a tendency to over-extract positively valenced or conventionally expected themes and biases inherited from training data, were mitigated by this comment-level human verification, although a residual anchoring effect of the model’s initial frame on the human coder cannot be fully excluded. Because the number of comments per participant varied widely, with a few prolific reporters contributing a disproportionate share, themes were summarized by the number of participants contributing at least one comment to each theme rather than by raw comment count, so the summary reflects the breadth of experience across participants rather than the reporting volume of a few. Time-series plots visualized the proportion of participants reporting positive versus negative changes over time.

### 2.8 Data management

All data were collected through the HIPAA-compliant SurveyMonkey platform. Data were de-identified and stored on a password-protected computer with encrypted transmission. A single password-protected file linked study IDs to participant identifiers and was accessible only to study staff; this file will be destroyed after publication of the study manuscript.

## 3 Results

### 3.1 Participant flow and sample characteristics

Participants were recruited along two parallel tracks: caregivers seeking to enroll a care recipient (*n* = 54 inquired) and self-response participants (*n* = 275 began the survey). Figure 1 presents the full participant flow. Across both tracks, the largest single source of attrition among otherwise-eligible volunteers was failure to submit the required physician approval letter (caregiver track: 1 of 9 who passed eligibility; self-response track: 39 of 58 who passed eligibility). This requirement, added to the protocol at the IRB’s request after the original study design was developed and submitted for review, was a substantial recruitment barrier and merits consideration in the design of future home-based TUS studies.

**FIGURE 1:**
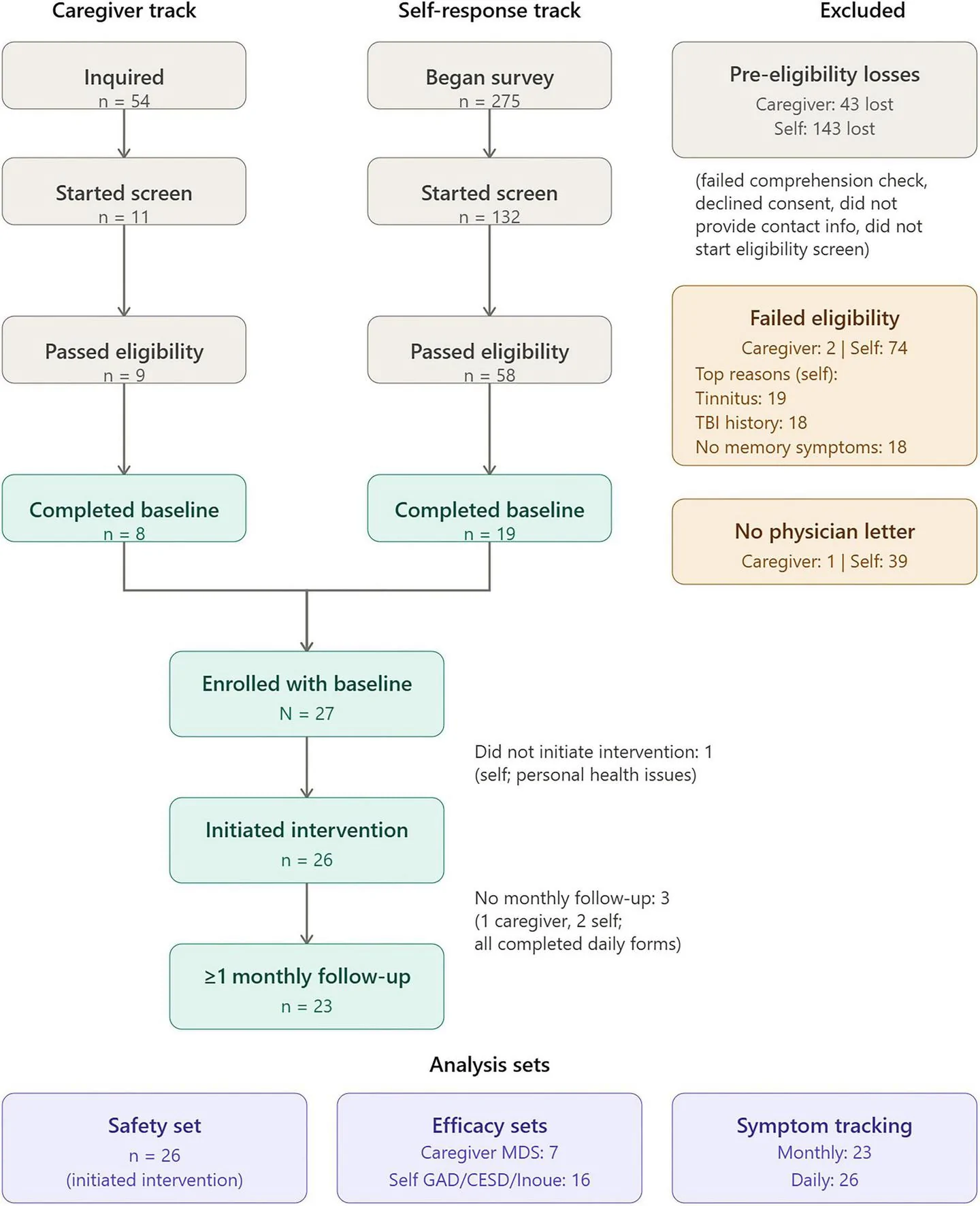
Participant recruitment.

Of 132 self-response participants who began screening, 74 (56%) failed at least one criterion, most commonly moderate-to-severe tinnitus (*n* = 19), traumatic brain injury (*n* = 18), or absence of self-reported cognitive symptoms despite interest (*n* = 18); the remainder failed on psychiatric history (*n* = 9), metal implants or medical devices (*n* = 6), or other criteria (*n* = 4). Among caregivers, 2 of 11 screened were excluded for the care recipient’s seizure or bleeding history.

A total of 27 participants completed the baseline assessment and were considered enrolled: 8 caregiver-administered (a caregiver completed all outcomes on behalf of a care recipient) and 19 self-administered. One self-administered participant did not initiate the intervention due to intervening personal health issues unrelated to the study and is excluded from all analysis sets. Of the remaining 26 who initiated treatment, 23 (88%) completed at least one monthly follow-up assessment; the 3 participants without monthly follow-up (1 caregiver-administered, 2 self-administered) all completed daily compliance reporting and contributed to safety and daily-symptom analyses.

Because inclusion criterion 5 permitted enrollment of individuals already using the device transcranially, the study baseline does not represent a pre-treatment state for all participants. One of the 26 participants who initiated treatment (3.8%) reported prior transcranial use, beginning approximately 5 weeks before baseline, with daily application of 5 to 10 min on the LOW setting at the temporal window. This participant was in the caregiver-administered group; no self-administered participant reported prior transcranial use.

Analysis sets were defined per aim. The safety set comprised all 26 participants who initiated the intervention. Efficacy sets required baseline plus at least one post-baseline monthly assessment on the outcome of interest: 7 for the caregiver-administered MDS 3.0 and 16 for the self-administered GAD-7, CESD-5, and Inoue Computerized Test Battery. Because the mixed-effects models use all available observations, they also included baseline data from the 18 self-administered participants (17 for the Inoue battery) who initiated treatment, which is the number reported in section “3.4.1 Self-Administered Outcomes.” Symptom-tracking analyses included 23 participants for monthly self-reported symptoms and 26 for daily symptom reports.

Baseline demographic and clinical characteristics of the enrolled sample (*n* = 26) are presented in Table 1, with stratification by administration mode (caregiver vs. self). The self-administered and caregiver-administered groups did not differ in sex or race but differed in age and socioeconomic status, with education trending toward significance: participants in the self-administered group were younger, reported lower socioeconomic status, and trended toward having more years of education (*p* = 0.051) than those in the caregiver-administered group. These differences reached nominal statistical significance; however, they should be interpreted with caution, as all three *p*-values were close to the 0.05 threshold and the caregiver-administered subgroup was small (*n* = 8), making the comparisons sensitive to individual participants.

**TABLE 1:** Baseline demographic characteristics by administration role, with group comparisons.

Characteristic	Self-administered (*n* = 18)	Caregiver-administered (*n* = 8)	Total (*n* = 26)	*p*
Age, years				0.039^a^
Mean (SD)	69.3 (11.1)	78.0 (5.5)	72.0 (10.5)	
Range	47–89	68–86	47–89	
Sex, *n* (%)			0.667^b^	
Female	13 (72.2)	5 (62.5)	18 (69.2)	
Male	5 (27.8)	3 (37.5)	8 (30.8)	
Race, *n* (%) †			0.529^c^	
European	17 (94.4)	8 (100.0)	25 (96.2)	
Native American	1 (5.6)	0 (0.0)	1 (3.8)	
African	1 (5.6)	0 (0.0)	1 (3.8)	
Latinx / Hispanic	1 (5.6)	0 (0.0)	1 (3.8)	
Education, years				0.051^a^
Mean (SD)	16.8 (2.6)	14.3 (3.0)	16.0 (2.9)	
Range	12–20	12–19	12–20	
Socioeconomic status (0–100) ‡				0.048^a^
Mean (SD)	73.9 (11.9)	84.8 (11.8)	77.2 (12.7)	
Range	47–89	66–100	47–100	

Continuous variables, mean (SD) with observed range; categorical variables, *n* (%). Group comparisons are unadjusted and should be read as descriptive and exploratory given the small caregiver-administered subgroup (*n* = 8), and non-significant results should not be read as evidence of equivalence.

*^a^*Wilcoxon rank-sum test.

*^b^*Fisher’s exact test.

*^c^*Fisher’s exact test on a person-level binary (identifies with ≥ 1 non-European category vs. European only); a category-by-category test was not performed because race was multiple-response with sparse cells. †Race was a multiple-response item; participants could select more than one category, so percentages may sum to > 100%. Categories with no responses in any group (Native Pacific Islander, Asian, Middle Eastern) are omitted; percentages are of valid cases within each column. ‡Self-rated on a 0–100 visual analog slider of perceived access to material and social resources (higher = greater access). The identical range for age and socioeconomic status in the self-administered group is coincidental.

### 3.2 Compliance and exposure

Device use compliance and exposure varied widely among the 26 participants and are summarized in Table 2. Across the cohort, participants accumulated approximately 23,400 min (≈390 h) of device use in total. On days when the device was used, participants generally delivered close to the prescribed 15-min daily dose (Table 2). Consequently, the substantial between-participant variation in cumulative exposure was driven primarily by how many days the device was used and how long participants remained in the study, rather than by underdosing within individual sessions. The device was used on a majority of enrolled days for most participants, though this proportion ranged widely (Table 2).

**TABLE 2:** Device-use compliance and exposure (*n* = 26).

Compliance / exposure variable	Mean (*SD*)	Median (*IQR*)	Range
Days in study	103 (72)	80 (45–142)	15–241
Days with daily assessment form completed	84 (73)	58 (33–113)	7–233
Days device used	65 (54)	51 (28–86)	5–188
Days used, % of enrolled days	60 (24)	64 (46–78)	10–100
Total device exposure, min	901 (784)	615 (388–1,154)	55–2,543
Minutes per use-day	13.4 (2.2)	14.1 (13.3–14.6)	6.9–15.2
Minutes per study-day	8.3 (3.7)	8.6 (5.2–11.0)	0.7–14.4

Exposure variables are right-skewed; both mean (SD) and median (IQR) are reported. Total device exposure and minutes per study-day were derived from self-reported daily logs (section “2.5 Compliance Operationalization”); days without a completed Daily Assessment Form were treated as zero exposure. Minutes per use-day = total device minutes ÷ days device used (target 15 min). Minutes per study-day = total device minutes ÷ days in study. Days used (%) = days device used ÷ days in study.

Engagement spanned the full possible range. Classifying participants by the proportion of enrolled days on which the device was used, 11 (42%) were high engagers ( ≥ 70% of days), 9 (35%) were moderate engagers (40–69%), and 6 (23%) were low engagers ( < 40%). Consistent with exposure being governed by participation rather than session dose, total device minutes were strongly correlated with days in the study (Pearson *r* = 0.83, *p* < 0.001; Spearman ρ = 0.82). This is the structural dose–duration relationship anticipated for the rolling-enrollment design (section “2.5 Compliance Operationalization”), and it motivates the sensitivity analysis substituting mean minutes per study day for total minutes (section “2.7 Statistical Analysis”).

Two participants adopted the authorized alternative schedule of two 7.5-min sessions per day, using it on the large majority of their device-use days (92% and 95%); the remaining participants predominantly used the standard three 5-min sessions, with only occasional longer sessions. This pattern supports the Daily Assessment Form coding in which a reported session longer than 5 min was treated as the protocol-authorized 7.5-min alternative (section “2.5 Compliance Operationalization”).

### 3.3 Aim 1 - safety

Across the 26 participants, drawn from 2,174 daily assessments and supplementary email and telephone correspondence, no serious adverse events and no unanticipated adverse events occurred; all reported events were mild and transient (Table 3). The only events assessed as related or possibly related to the intervention were headache, reported by four participants (15.4%), and jaw/teeth clenching, reported by one participant (3.8%). Headaches were mild, in one case a dull ache rated 3 on a 10-point scale beginning a few minutes into a session. These were frequently associated with and responsive to increased hydration, and resolved or improved in every case with available follow-up. The single report of jaw/teeth clenching was provided through follow-up correspondence after the participant had already withdrawn; the same participant described a transient side-of-head ache and a single use of the device above the protocol-specified LOW intensity, and the clenching diminished after discontinuation.

**TABLE 3:** Adverse events (*n* = 26).

Adverse event	Participants, *n* (%)	Assessed related	Severity / outcome / attribution
Headache	4 (15.4)	Related/possibly	Mild; during or shortly after sessions; frequently hydration-responsive; resolved or improved
Jaw/teeth clenching	1 (3.8)	Related/possibly	Mild; reported via follow-up correspondence; diminished after discontinuation; co-occurred with a single use above the LOW setting.
Tinnitus^a^	1 (3.8)	Unrelated	Pre-existing and intermittent; transient in-study exacerbation; resolved
Sleep disturbance	2 (7.7)	Unrelated	Mild; one participant on sleep medication
Low mood / irritability	2 (7.7)	Unrelated	Long-standing and medicated, or attributed to external stressors
Fatigue / sleepiness	3 (11.5)	Unrelated	Mild
Visual (eye strain / blurriness)	2 (7.7)	Unrelated	Includes a pre-existing cataract
Neck discomfort	1 (3.8)	Unrelated	Mild; attributed to other factors
Tremor / edginess	1 (3.8)	Unrelated	Pre-existing; also present during the off-week
**Serious adverse events**	**0**	–	–
**Unanticipated adverse events**	**0**	–	–

Counts are participant-level; a participant may appear in more than one row. Events were drawn from the Daily Assessment Form and study email/telephone correspondence and de-duplicated per participant. All reported events were mild; none met criteria for a serious adverse event: any event resulting in death, a life-threatening condition, inpatient hospitalization or prolongation of existing hospitalization, persistent or significant disability or incapacity, or requiring intervention to prevent permanent impairment; none were unanticipated: any event whose nature, severity, or frequency was not described in the study protocol, the informed consent documents, or the existing transcranial ultrasound literature for this device and population.

^a^Note on tinnitus. The participant who reported tinnitus had a pre-existing, intermittent, and self-limiting form that did not meet the study’s moderate-to-severe exclusion threshold; this was confirmed against their screening record, in which moderate-to-severe tinnitus was not reported. The in-study exacerbation was transient and resolved. Relatedness reflects the investigator’s assessment. Events attributed to pre-existing conditions, intercurrent illness, or external circumstances were classed as unrelated (see text). Reports of transient worsening of memory or cognition were interpreted as underlying-disease fluctuation and are analyzed under Aim 3, not as adverse events.

The remaining reported negative changes were assessed as unrelated to the intervention and attributable to pre-existing conditions, intercurrent illness, or external circumstances (Table 3): tinnitus (pre-existing, with a transient exacerbation that resolved; *n* = 1), sleep disturbance (*n* = 2), low mood or irritability (long-standing or stress-related; *n* = 2), fatigue or sleepiness (*n* = 3), visual symptoms including a pre-existing cataract (*n* = 2), neck discomfort (*n* = 1), and tremor or edginess (pre-existing; *n* = 1). Two participants had intercurrent infections: a cold, and a COVID-19 infection that prompted withdrawal, unrelated to the device. Transient worsening of memory or cognition was interpreted as an underlying disease fluctuation and addressed under Aim 3.

No discontinuations were attributable to adverse events. The only withdrawal associated with a health event was the COVID-19 infection, which was unrelated to the device; the participant who later reported mild jaw/teeth clenching had already discontinued for unrelated reasons and did not identify those symptoms as the cause. A small number of participants briefly paused treatment in response to mild symptoms and resumed without recurrence. Overall, the safety profile was consistent with prior transcranial-ultrasound literature (Sarica et al., 2022).

### 3.4 Aim 2 - efficacy

#### 3.4.1 Self-administered outcomes

Eighteen self-administered participants contributed to the Aim 2 efficacy analyses (GAD-7 and CESD-5, *n* = 18; Inoue Computerized Test Battery, *n* = 17 at baseline). The number of participants contributing data declined across successive monthly visits (from 18 at baseline to 3 by the eighth visit), reflecting the rolling-enrollment design and variable participation duration; mixed-effects models used all available observations under a missing-at-random assumption.

Anxiety (GAD-7). Self-reported anxiety decreased from a baseline mean of 5.83 (*SD* 3.81) toward the lower end of the scale across follow-up (Visit 8 mean 2.00, *SD* 2.00). In the mixed-effects model, the effect of time was negative but did not reach statistical significance (β = –0.197 per visit, 95% CI –0.394 to –0.005, *p_FDR* = 0.074), indicating a downward trend in anxiety symptoms that was not statistically reliable in this small sample.

Depression (CESD-5). Self-reported depressive symptoms showed a statistically significant decline over time (β = –0.908 per visit, 95% CI –1.53 to –0.29, *p_FDR* = 0.016). CESD-5 scores were multiplied by four to align with the 0–60 metric of the 20-item CES-D (conventional screening cutoff ≈ 16); on this scale, the mean fell from 20.0 (*SD* 12.7) at baseline, just above the screening cutoff, to 11.2 (SD 10.4) by the sixth visit. This was the only self-administered outcome to show a statistically significant change over the course of treatment.

Cognition (Inoue Computerized Test Battery). Total Inoue scores were near the top of the measurement range at baseline (mean 14.23 of a possible 15, *SD* 1.03) and remained stable across follow-up, with no statistically significant change over time (β = –0.161 per visit, 95% CI –0.36 to 0.04, *p_FDR* = 0.133). The pronounced ceiling at baseline left little room to detect improvement on this objective measure, and the single low value at Visit 7 (mean 11.00, *SD* 5.35, *n* = 4) reflects one low-scoring participant in a small cell rather than a population-level decline.

Covariate effects. Neither age nor cumulative device-use time was significantly associated with any self-administered outcome (all *p* ≥ 0.84). The absence of a relationship between total minutes of device use and the outcome indicates no detectable dose–response relationship across the observed range of exposure in this sample.

Descriptive statistics by visit and mixed-effects model estimates are presented in Supplementary Table 1; outcome trajectories over time are shown in Figure 2.

**FIGURE 2:**
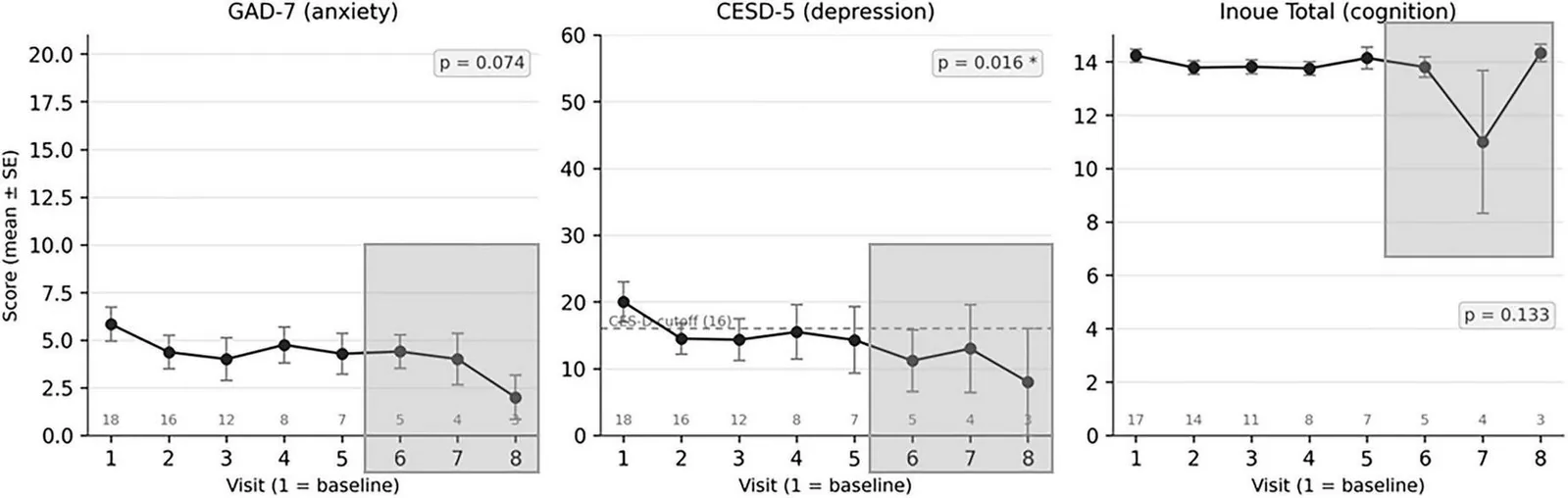
Self-administered outcomes over time. Error bars = standard error of the mean. Numerals along the x-axis indicate the number of participants contributing at each visit. CESD-5 scores multiplied by four to match the 20-item CES-D scale (0–60); dashed line marks the conventional screening cutoff of 16. Visits contributing fewer than one third of the baseline sample (*n* = 6) are shaded and their markers de-emphasized, as these estimates rest on few participants and are unstable. * = significant after FDR correction.

#### 3.4.2 Caregiver-administered outcomes

Eight care recipients had cognitive and mood outcomes rated by a caregiver using the MDS 3.0 (Sections C and D), contributing 42 participant-visit observations (41 for long-term memory) across up to nine monthly assessments. As in the self-administered group, the number contributing declined over follow-up (from 8 at baseline to 1 by the final visit); mixed-effects models used all available observations.

No caregiver-administered outcome changed significantly over time after FDR correction. Per-visit slopes were small for short-term memory (C07), long-term memory (C08), memory/recall ability (C09), decision-making (C10), and mood (PHQ-9), and none survived correction (all *p*_FDR ≥ 0.33). The largest nominal effect was a slight worsening in decision-making (C10: β = +0.05 per visit, raw *p* = 0.067, *p*_FDR = 0.33), a direction consistent with expected disease progression rather than benefit. Given the small subgroup (*n* = 8) and the approximation involved in fitting linear models to binary and ordinal outcomes, these findings are reported descriptively here; model estimates appear in Supplementary Table 2.

Neither age nor cumulative device-use time was associated with any outcome in the primary models (all raw *p* ≥ 0.26). The GEE sensitivity analysis confirmed the absence of any time effect on every outcome; a nominal GEE association between device-use minutes and decision-making and mood was not corroborated by the mixed models and is attributable to the known anti-conservatism of robust standard errors with few clusters (here, eight), and is therefore not interpreted as evidence of dose-response.

#### 3.4.3 Sensitivity analysis

Two sensitivity analyses were conducted for the Aim 2 efficacy models.

##### 3.4.3.1 Mean minutes per study day

Because total device-use minutes were strongly correlated with days in the study (section “3.2 Compliance and Exposure”), the Aim 2 models were re-fitted with mean minutes per study day in place of total minutes, separating cumulative dose from time in study. Conclusions were unchanged in both groups. Among self-administered outcomes, the CESD-5 decline remained significant (β = –0.91 per visit, *p* = 0.005), the GAD-7 and Inoue remained non-significant, and the mean-minutes covariate was itself non-significant in all three models (all *p* ≥ 0.25), as total minutes had been. Among caregiver-administered outcomes, no MDS measure changed significantly (the largest effect was decision-making, *p* = 0.085), and the mean-minutes covariate was non-significant throughout (all *p* ≥ 0.49). The compliance covariate thus showed no association with outcome under either operationalization, indicating the null dose findings were not an artifact of the dose-duration relationship inherent to the rolling-enrollment design.

##### 3.4.3.2 Generalized estimating equations

Because several outcomes departed from normality, GEE models (exchangeable working correlation, robust standard errors) were fitted as a robustness check on the self-administered Aim 2 outcomes. The GEE results reproduced the mixed-model conclusions: CESD-5 declined significantly (β = –0.90, *p* = 0.003), GAD-7 and Inoue did not change, and the device-use covariate was non-significant throughout (all *p* ≥ 0.42). Unlike the caregiver-administered GEE (section “3.4.2 Caregiver-Administered Outcomes”), in which robust standard errors produced a spurious device-use association given the small number of clusters (*n* = 8), the self-administered GEE, with a more adequate cluster count (*n* = 18), showed no such artifact. The mixed-effects models are retained as primary, with conclusions unchanged.

### 3.5 Aim 3 - subjective cognitive symptom trajectories

#### 3.5.1 Monthly self-report symptoms

The monthly seven-item symptom assessment and its composite were analyzed separately for the self-administered (*n* = 16) and caregiver-administered (*n* = 7) groups, reflecting differences in measurement mode (self- vs. proxy-report) and the greater baseline symptom burden among caregiver-rated recipients (see Figure 3).

**FIGURE 3:**
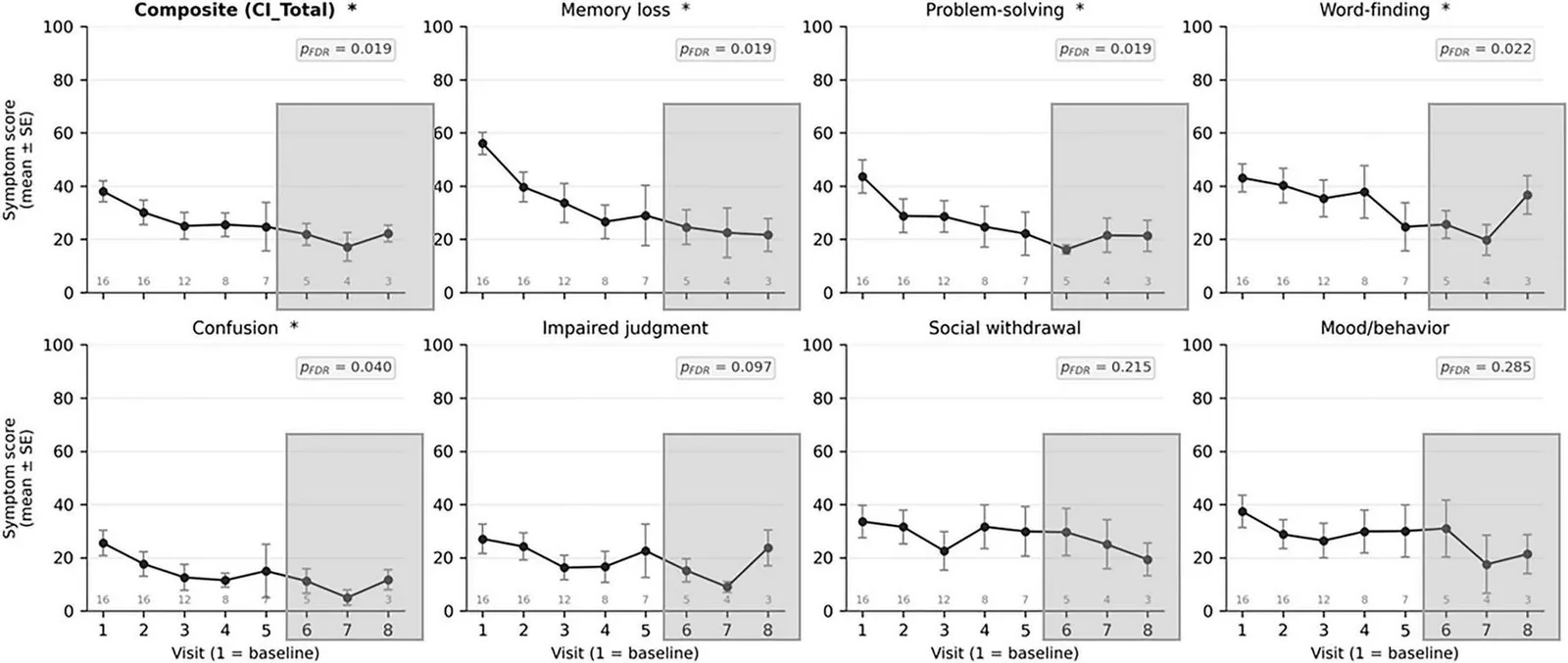
Monthly self-reported symptom trajectories - self-administered group. Self-administered group (*n* = 16 at baseline). Higher scores = greater symptom burden; downward slope = improvement. * = significant after FDR correction. Numerals along the *x*-axis = participants contributing per visit. Visits contributing fewer than one third of the baseline sample (*n* = 6) are shaded and their markers de-emphasized, as these estimates rest on few participants and are unstable.

Among self-administered participants, self-reported symptoms decreased significantly over time across most domains. After FDR correction, significant reductions were observed for memory loss (β = –3.79 per visit, 95% CI –6.36 to –1.22, *p_FDR* = 0.019), problem-solving and planning difficulty (β = –3.16, 95% CI –5.42 to –0.90, *p_FDR* = 0.019), the composite symptom score (β = –2.35, 95% CI –3.98 to –0.73, *p_FDR* = 0.019), word-finding difficulty (β = –2.91, 95% CI –5.11 to –0.70, *p_FDR* = 0.022), and confusion (β = –2.11, 95% CI –3.95 to –0.27, *p_FDR* = 0.040). Impaired judgment showed a non-significant trend toward improvement (β = –1.80, 95% CI –3.78 to 0.17, *p_FDR* = 0.097), while social withdrawal (*p*_FDR = 0.215) and mood or behavior changes (*p*_FDR = 0.285) did not change significantly. All significant effects were in the direction of symptom reduction. Descriptively, the composite fell from 38.0 (*SD* 15.6) at baseline to the low 20 s over follow-up. Neither age nor cumulative device-use time was associated with any symptom outcome (all *p* ≥ 0.26).

In the caregiver-administered group, no symptom showed a significant change over time; all effects were non-significant after FDR correction (all p_FDR ≥ 0.84), with small slopes of inconsistent direction. The caregiver-rated composite remained elevated across follow-up (61.0 at baseline to the low-to-mid 50 s). Age and device-use minutes were likewise unrelated to any outcome.

As in the Aim 2 efficacy analyses, the pattern diverged sharply by group: self-administered participants reported broad symptomatic improvement over the course of treatment, whereas caregiver-rated recipients did not. Model estimates and descriptive statistics for all eight outcomes in both groups are provided in Supplementary Table 3.

*Post-hoc* analyses revealed that baseline symptom scores differed markedly between the two groups. Memory loss, word-finding difficulty, confusion, problem-solving and planning difficulty, and impaired judgment, together with the composite index, were significantly higher at baseline in the caregiver-administered group (all *p* ≤ 0.022; Figure 4), consistent with the greater symptom burden among caregiver-rated recipients. The only two items that did not differ between groups were mood or behavior changes (*p* = 0.85) and social withdrawal (*p* = 0.40). These were also the two items that showed no significant change over time in the self-administered group (Figure 3).

**FIGURE 4:**
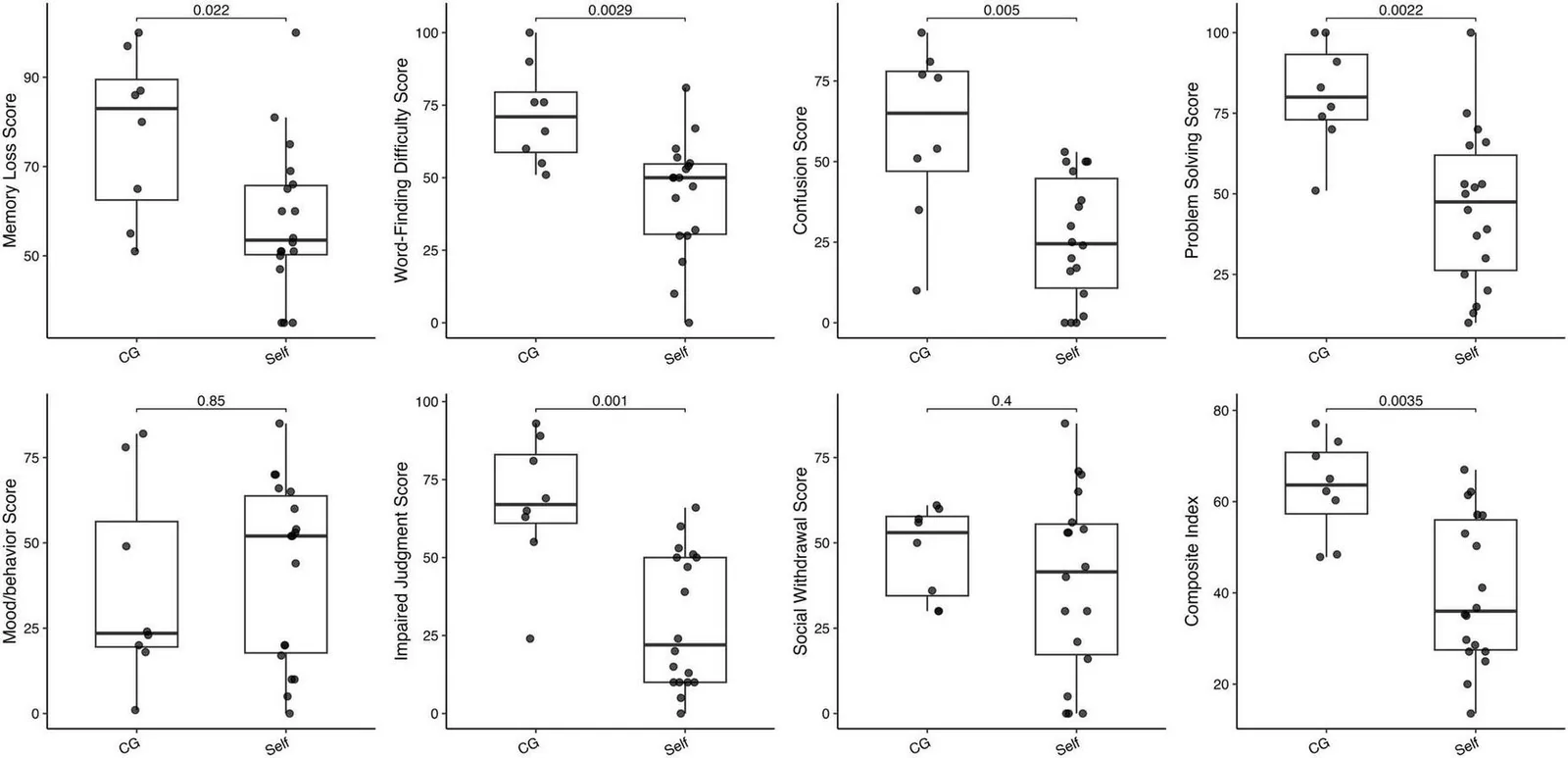
Comparison of baseline self-reported symptoms between the self and caregiver administered groups. CG, caregiver administered group; Self, self-administered group. *P*-values are shown above each comparison and were calculated using two-sided Wilcoxon rank-sum tests.

#### 3.5.2 Daily symptom reports

Participants submitted 2,174 Daily Assessment Forms over the course of the study (a median of 58 forms per participant). Daily symptom-change reports were summarized at the participant level to avoid weighting results toward participants who completed more forms. Over the course of the study, 20 of 26 participants (76.9%) reported at least one positive symptom change, and 14 of 26 (53.8%) reported at least one negative change; 12 participants (46.2%) reported no negative change at any point, and 6 (23.1%) reported no positive change (See Figure 5). Within participants, the proportion of completed daily forms indicating a positive change (median 8.1%, IQR 1.4–20.1%, range 0–44.6%) generally exceeded the proportion indicating a negative change (median 1.1%, IQR 0–5.8%, range 0–21.1%), indicating that for most participants daily reports of improvement were more frequent than reports of worsening, though both were uncommon relative to reports of no change.

**FIGURE 5:**
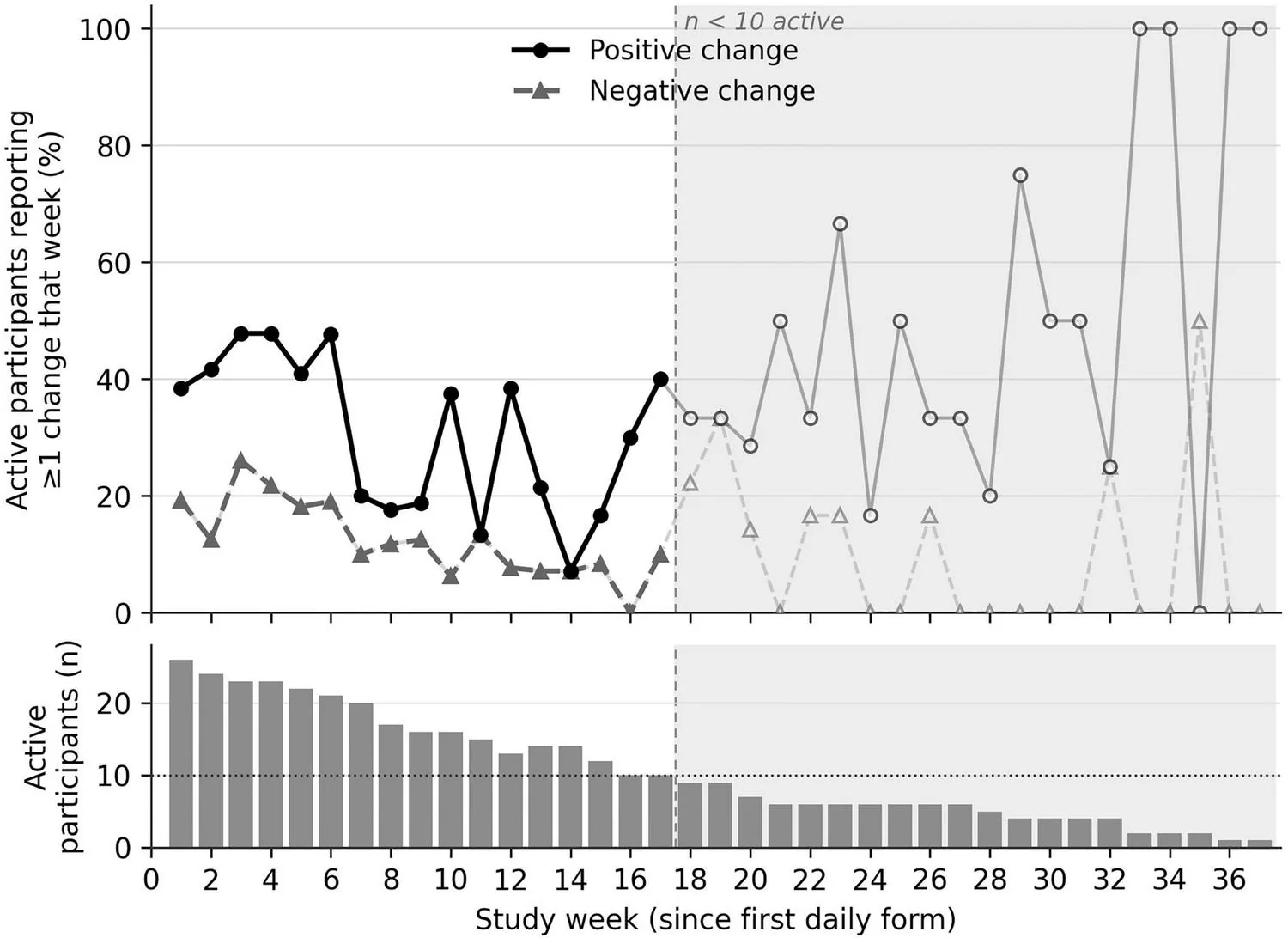
Daily symptom-change reports over time. Top*:* percentage of active participants (those submitting ≥ 1 Daily Assessment Form in a given week) who reported at least one positive change (solid line, filled circles) or at least one negative change (dashed line, filled triangles), by study week relative to each participant’s first daily form. *Bottom:* number of active participants contributing data each week (the denominator). The shaded region (study weeks ≥ 18) marks weeks in which fewer than 10 participants were active; markers there are faded and open because these estimates rest on small, declining samples and should be interpreted with caution. Positive reports exceeded negative reports in essentially every week.

#### 3.5.3 Qualitative theme analyses of daily symptom reports

Open-ended daily comments (294 positive, 60 negative) were analyzed thematically (section “2.7 Statistical Analysis”); full themes, participant counts, and representative quotes appear in Supplementary Table 4. Positive comments clustered in three domains. Cognitive themes were most common, spanning memory and recall (9 participants) and mental clarity or focus (8 participants), with one participant reporting that “brain fog is gone.” Affective themes were equally prominent, with improved mood (8 participants) and a sense of calm or reduced anxiety (8 participants). Functional themes described renewed engagement in activities and hobbies (8 participants), several with striking specificity: one participant “read a book for 2 h,” something “not done for the past 2 years,” and another “got my kayak out” after a year of disinterest. Less frequent themes included improved energy, social engagement, sleep, and dream recall. Among caregiver-rated recipients, one previously withdrawn care recipient began initiating activity and asking others “How are you doing?,” described as “a first in years.”

Negative comments were fewer and dominated by fluctuations in the underlying cognitive condition (fog, confusion, or worsening memory; 6 participants), together with the mild, transient physical symptoms detailed under Aim 1 (most commonly headache; 4 participants). A small but notable subset described a perceived decline during the scheduled one-week off periods, for example, “a decline in recall/mood since beginning my first off week.”

A cross-cutting feature of both positive and negative comments was frequent causal hedging. Participants regularly questioned whether changes were attributable to the device; one noted, “it is hard to determine what is changing,” and, in several cases, explicitly attributed changes to non-device factors such as new medications, intercurrent illness, weather, or life stress (8 participants). This epistemic caution, while limiting causal inference, lends credibility to the comments as candid self-observation rather than uniform expectancy, and reinforces the need for a controlled comparison.

## 4 Discussion

### 4.1 Summary of findings

This open-label pilot evaluated the safety, preliminary efficacy, and symptom trajectories of home-based low-intensity transcranial ultrasound in adults with memory and cognitive difficulties, across three protocol-specified aims. For Aim 1, the safety hypothesis was supported: across 26 participants and more than 2,000 daily assessments, no serious or unanticipated adverse events occurred, and the only related events were mild, transient headache and a single report of jaw clenching, consistent with the TUS safety literature.

For Aim 2, the efficacy hypothesis was partially and selectively supported. Self-administered participants showed a significant decline in depressive symptoms, while anxiety trended downward non-significantly, and the objective cognitive battery, near ceiling at baseline, did not change. Caregiver-rated cognitive and mood outcomes did not change on any measure, and device exposure was unrelated to outcomes, with these patterns holding across the mean-minutes and GEE sensitivity analyses.

For Aim 3, the symptom-tracking hypothesis held within the self-report data but not beyond it. Self-administered participants improved on most monthly symptom domains; their daily reports favored improvement over worsening throughout, and qualitative themes clustered in the cognitive, affective, and functional domains that the intervention was hypothesized to influence. As in Aim 2, this improvement was specific to self-raters. Across all three aims, then, TUS was safe and accompanied by broad self-reported improvement that was absent from the objective measure and the caregiver ratings, a divergence that frames the questions below. We highlight that because the objective cognitive battery and the caregiver ratings did not change, efficacy cannot be inferred from the self-reported improvements observed here. In this uncontrolled, open-label design those reports remain fully compatible with expectancy, placebo response, and other non-specific effects.

### 4.2 Safety profile

The safety profile was favorable and consistent with the transcranial ultrasound literature. No serious or unanticipated events occurred, and all reported events were mild and transient. The only events judged related or possibly related to the intervention were headache and, in one participant, jaw or teeth clenching, both well within the spectrum of transient effects cataloged for low-intensity ultrasound neuromodulation (Miller et al., 2012; Radjenovic et al., 2022; Sarica et al., 2022).

The headache rate here (15.4% of participants) exceeds the pooled 3.4% transient-event rate reported in the Sarica et al. (2022) review, but this reflects the study design rather than a greater hazard. The studies Sarica aggregated were largely single-session or short-course interventions in supervised settings. In contrast, the present study involved repeated self-administered home use over weeks to months with active daily symptom solicitation, conditions that increase both the opportunity for transient events and their ascertainment. Headache is among the most frequently reported TUS effects, so its emergence here is expected. In addition, the estimate includes 4 of 26 participants, so its precision is low, with a Wilson 95% confidence interval of approximately 6% to 34%, an interval that overlaps rates reported in individual TUS studies. The participant-level percentage accumulates risk across an unusually long exposure window, up to three sessions daily over weeks to months and spanning more than 2,000 assessed days, so the corresponding per-session incidence is very low relative to the largely single-session exposures behind the pooled 3.4% figure. Headache also has a substantial background prevalence in the general adult population, and no pre-treatment headache run-in was collected, so some events likely reflect base rates rather than device effects. The headaches were mild, frequently coincided with and responded to increased hydration, and resolved or improved in all cases, more consistent with non-specific contributors than with any neurotoxic effect. One benign contributor is worth noting: the temporal-window technique engages the temporalis and masseter muscles, and the device instructions direct users to clench the jaw to confirm placement, which could plausibly contribute to both tension-type headache and the jaw clenching reported by one participant, locating these events in the application technique rather than the ultrasound itself. This interpretation is speculative but testable, and addressable through refined placement guidance.

Overall, the absence of serious or unanticipated events during unsupervised home use supports advancing the intervention to a controlled trial. However, this is tempered by the open-label, self-report nature of adverse-event ascertainment and the unblinded relatedness judgments.

### 4.3 Preliminary efficacy

The two groups showed markedly different trajectories. In the self-administered group, self-reported symptoms improved significantly over time, with memory loss, word-finding, problem-solving, the cognitive-impairment composite, and depressive symptoms all declining, and confusion at the significance threshold and anxiety trending. The objective cognitive battery, which was near ceiling at baseline, did not change. In the caregiver-administered group, no outcome changed significantly, and cumulative exposure predicted change in neither group.

The divergence is most likely explained by expectancy rather than a specific treatment effect. The observed dissociation, with improvement confined to unblinded self-reports from the less-impaired participants who administered the device themselves and no change on the objective battery or on informant ratings of the more impaired recipients, is the characteristic signature of an unblinded subjective endpoint. Less-impaired self-raters are precisely the group most able to generate expectancy-driven self-reports, whereas informant ratings are comparatively expectancy-resistant. Recruitment through networks of existing device users, which selects for participants favorably disposed to the intervention, further amplifies this concern (section “4.8 Strengths and Limitations”). We therefore treat placebo and expectancy response as the primary explanation of the group divergence pending sham-controlled data. A secondary, hypothesis-generating consideration is cognitive reserve and disease stage, which aligns with the baseline differences between the groups. Self-administered participants were younger and more educated than the caregiver-rated recipients (Table 1) and were substantially less impaired. In a *post-hoc* comparison, baseline symptom scores were significantly higher in the caregiver-administered group on five of the seven items and on the composite index (Figure 4), confirming that the two groups began from markedly different levels of impairment on the same instrument. Higher educational attainment is a well-established proxy for cognitive reserve, associated with greater resilience to decline and greater capacity to maintain function despite neuropathology (Stern, 2012). The older, more severely impaired recipients may have been beyond the window in which a low-intensity intervention, or the instruments used to detect it, could register benefit. On this account, any true benefit would have concentrated in younger, higher-reserve, less-impaired individuals, nominating mild cognitive impairment or earlier-stage disease as a more promising target than advanced dementia. Without a control condition we cannot weigh this explanation against expectancy, so we raise it only as a hypothesis for future testing.

The same comparison points to a more specific, domain-level pattern. The two items that did not separate the groups at baseline, mood or behavior changes, and social withdrawal, were also the only two that did not improve over time in the self-administered group, suggesting that both the between-group difference and the within-group change concentrated in the cognitive domains rather than the affective or behavioral ones. This observation is necessarily tentative, since the two analyses share participants and instruments, and the alignment could reflect a common measurement property or chance in a small sample.

This contrast is confounded, however. The groups differed simultaneously in age, education, baseline severity, and sample size, so the divergence cannot be attributed to any single factor. In particular, the caregiver group’s small size (*n* = 8) and greater variability limited power, so its null result should not be read as an established absence of effect. The differential pattern is therefore best treated as hypothesis-generating, nominating cognitive reserve and disease stage as moderators to test prospectively.

### 4.4 Self-reported symptom trajectories

Across the study, participants reported daily improvement more often than worsening: positive change reports outnumbered negative ones at the participant level; most participants reported at least one positive change; nearly half reported no negative change at any point; and improvement exceeded worsening in essentially every adequately powered week. This daily pattern mirrored the monthly data, where the self-report composite and four items declined significantly among self-administered participants (section “3.5.1 Monthly Self-Report Symptoms”). The convergence of the daily and monthly streams, collected on different schedules, strengthens the impression of a consistent within-person trajectory of perceived improvement, and the qualitative themes gave it content, concentrating on the cognitive, affective, and functional domains the intervention was hypothesized to influence.

A further consideration is the expected natural trajectory over the study window. In mild cognitive impairment and early Alzheimer’s disease, decline on standard cognitive instruments accrues gradually, on the order of a few points per year, so comparable untreated individuals would be expected to show little measurable change over the three to six months most participants contributed (Han et al., 2000; Bennett et al., 2002). Against a roughly flat expected trajectory, the improvement in the self-administered group cannot be attributed to the halting of the anticipated decline and is equally consistent with regression to the mean, expectancy, or ordinary fluctuation. This reasoning is necessarily approximate, since our heterogeneous, self-identified sample does not map onto any single natural-history cohort, and the seven-item symptom sliders are not a validated instrument with an established rate of change.

These convergent self-reports are encouraging but cannot support any inference of efficacy in an open-label design. Daily change-since-last-application reports are especially susceptible to expectancy effects, and the predominance of positive reports could reflect a placebo response, regression to the mean, or ordinary day-to-day variability rather than an ultrasound effect. The consent and information materials described the intervention as exploratory and research-focused, distinct from standard care, and stated that potential cognitive or quality-of-life benefits might result but were not guaranteed. Nonetheless, because the materials described the device in terms of its potential to improve memory and cognition and recruitment drew on networks of existing device users, a favorable disposition toward the intervention cannot be excluded.

Some features weigh against, without excluding, a purely non-specific account. Participants frequently hedged their causal attributions and often credited non-device factors such as new medications, illness, weather, or stress, which were more consistent with candid self-observation than with uniform expectancy. Several reports described concrete behavioral changes, such as resuming reading or a long-abandoned hobby, or in one caregiver-rated recipient, initiating conversation after years of withdrawal, that are less readily explained by momentary expectancy than global ratings of feeling better. And the subset who perceived decline during scheduled off-weeks describes an on-off pattern at least consistent with a treatment-linked effect, though equally compatible with expectancy tracking the schedule. None is decisive. The validated instruments provide a more rigorous test, and the daily and qualitative data are best read as complementary, identifying candidate outcome domains, memory, mood, and everyday functional engagement, for the controlled trial this pilot was designed to inform.

### 4.5 Adherence and dose-response

Adherence to the self-administered protocol was variable but informative. On days participants engaged the device, they generally delivered close to the prescribed 15-minute dose, so the wide between-participant variation in cumulative exposure was driven mainly by how many days the device was used and how long participants remained enrolled, rather than by under-dosing within sessions. Within-session dosing was therefore close to target, while between-day adherence was the limiting factor. Whether this is a sufficient basis for an efficacy trial built around unsupervised home administration is addressed in section “4.6 Recruitment and Feasibility”. Cumulative exposure was not a significant predictor of change in any model. This null dose-response is open to several interpretations: in an uncontrolled design, it is consistent with non-specific effects, a ceiling at the doses achieved, or limited power in a small, heterogeneous sample. Interpretation is further limited by a self-reported compliance measure that could not verify placement or technique, reinforcing the need for objective, device-based logging of session occurrence, duration, placement and intensity in future controlled studies.

The intensity of stimulation is a further parameter left unexamined here. This study fixed intensity at the device’s LOW, sub-thermal setting to remain within the established safety window, so whether the modality has an intensity-dependent effect, and where any benefit might plateau, is unknown. Systematic variation of intensity, alongside duration and frequency, within safe limits is warranted in future controlled work to characterize the dose-response surface.

### 4.6 Recruitment and feasibility

As a feasibility study, this pilot surfaced practical lessons, foremost among them recruitment. Although we aimed to enroll up to 100 participants, several factors constrained accrual. Most consequentially, our IRB appropriately required documented physician approval before enrollment, a safeguard we endorse for an investigational device used outside its cleared indications. However, obtaining a physician letter proved a substantial barrier for many interested individuals. Despite explicit statements that a US Pro2000 device was required, many interested people did not own one. Enrolling two distinct populations, self-administering participants and caregivers reporting on a recipient, further added heterogeneity in age, impairment, and measures.

Several of these obstacles are addressable. Conducting future studies in clinical settings, with treating physicians as investigators, would remove the physician-letter bottleneck and streamline eligibility. Partnering with residential care facilities would support the enrollment of more impaired patients and enable trained staff to administer the intervention consistently, thereby improving adherence. Separating the self- and caregiver-administered populations into distinct studies, each with measures matched to their respective impairment levels, would reduce heterogeneity.

The adherence data speak directly to whether an efficacy trial can be designed around home self-administration. Only 42% of participants were high engagers, using the device on 70% or more of enrolled days; 23% used it on fewer than 40% of days; and the mean delivered dose was roughly 55% of target, at 8.3 of a prescribed 15 min per study-day. Adherence was moreover entirely self-reported, with days lacking a daily form coded as zero exposure and placement or technique unverifiable. This level of real-world adherence is not, on its own, a sufficient foundation for a definitive efficacy trial of unsupervised home use. Such a trial should either embed delivery in supervised or facility-based settings or, if home administration is used, incorporate device-based usage logging, an adherence run-in period before randomization, and a protocol-specified minimum-dose criterion, with adherence itself analyzed as a co-primary feasibility endpoint. These clinical and care-facility partnerships are the most direct route to the enrollment, adherence, and physician pre-approval that a definitive trial will require.

### 4.7 Implications and future directions

The intervention examined here remains exploratory and is not an established therapy. The present results do not demonstrate efficacy in a sample of this size; the study was designed to assess feasibility, safety, and preliminary signal. For that reason, we have declined requests to distribute the protocol as a treatment, presenting it as an open research question until adequately controlled trials are completed. Several features nonetheless make it attractive for continued investigation: the device is inexpensive, portable, and home-deliverable, it was generally well tolerated, and no serious or unanticipated events emerged. In contrast to MRI-guided focused ultrasound and other resource-intensive approaches, this accessibility would make the modality unusually scalable for a condition as prevalent as cognitive impairment, if efficacy were established.

The pilot’s clearest contributions are to the design of the planned double-blind, sham-controlled trial. That type of trial is unusually tractable: because an identical inert session can be delivered simply by leaving the device unpowered, a sham arm requires no specialized equipment and supports robust blinding of participants and assessors, directly addressing the expectancy and regression-to-the-mean concerns that limit the present findings. The preliminary estimates here provide the variance and trajectory information needed to power such a trial, and several lessons follow. A future trial should focus on mild cognitive impairment or early-stage disease, in whom there is room to change, and pair subjective measures with objective cognitive and, ideally, biological or imaging endpoints, because the signals are concentrated in self-reported symptoms among less-impaired, higher-reserve participants. Instruments with demonstrated sensitivity to change and adequate range in mildly impaired samples, such as the Montreal Cognitive Assessment, or a remotely deliverable computerized battery, should replace brief screening batteries prone to ceiling effects. The dense daily assessment proved feasible and could serve as a sensitive, ecologically valid secondary outcome once blinded. Because exposure was unrelated to outcome, the optimal dose, intensity, and schedule remain unknown and should be examined directly with a fixed-duration design that avoids the rolling-enrollment dose-duration confound. Enrolling a single, well-characterized population rather than combining administration modes would reduce heterogeneity, and the trial should be embedded in clinical or residential-care settings to overcome the recruitment and adherence barriers encountered here. In parallel, controlled animal and *in vitro* models are needed to test the hypothesized microtubule-resonance mechanism under conditions that human studies cannot isolate.

### 4.8 Strengths and limitations

Several limitations bear on interpretation. The open-label, within-participant design without randomization or control limits causal inference; observed changes cannot be unambiguously attributed to ultrasound rather than to placebo, regression to the mean, natural fluctuation, or expectancy. Eligibility was determined by self-report rather than clinical confirmation, which may introduce heterogeneity in the underlying condition. Thus, the self-administered subgroup is better characterized as experiencing subjective cognitive decline, or mild impairment not detected by a brief screening battery, than as having objectively verified cognitive impairment. The objective cognitive battery showed a pronounced ceiling at baseline, rendering it insensitive to change in this largely mildly impaired sample and leaving self-report as the only modality capable of registering improvement in that group. The intervention was self-administered at home, and adherence to the prescribed temporal window placement could not be verified.

Compliance was measured solely through self-report, with no objective verification of session occurrence, duration, placement, or intensity setting. Acoustic output was not independently calibrated, and the intracranial intensity actually delivered is unknown, because transmitted energy depends on transducer output, coupling quality, and individual skull thickness and attenuation at the temporal window. Reported exposure therefore indexes nominal application time rather than delivered acoustic dose, which constrains any dose interpretation, limits reproducibility, and complicates comparison with other TUS studies. A literature-based estimate frames the likely exposure. Skull attenuation is strongly frequency dependent and rises with frequency, so at the 1 MHz operating frequency of this device the transmitted fraction of incident intensity is expected to be low, and it varies several-fold with local skull thickness and density across sites and specimens (White et al., 2006). The adequacy of the temporal acoustic window itself also varies widely between individuals, is reported to be inadequate in roughly 8% to 20% of adults (Marinoni et al., 1997; Chan et al., 2023), and becomes more common with advancing age and in women. Our sample had a mean age of 72 years and was 69% female, the demographic in which inadequate temporal windows are most frequent, so a non-trivial proportion of participants may have received little or no intracranial exposure. Actual brain exposure was therefore unknown and is likely to have varied considerably across participants, which also limits the interpretation of the dose-response analyses. This uncertainty is conservative with respect to safety, since exposure at the transducer face was already well below applicable therapeutic and diagnostic limits (section “2.3 Intervention”) and in-situ exposure can only have been lower. Future trials should include independent hydrophone calibration of device output together with estimation or measurement of transcranial transmission. The rolling-enrollment design produces a structural relationship between cumulative dose and time in study, which the sensitivity analysis addressed but cannot fully eliminate.

Recruitment through networks of existing device users may have introduced selection bias toward individuals favorably disposed to the intervention. Relatedly, participants could enroll while already using the device transcranially (inclusion criterion 5); for these individuals, baseline is not a pre-treatment assessment, their trajectories cannot be interpreted as treatment-initiation trajectories, and prior use could bias change estimates toward the null or reflect plateau effects; this applied to one caregiver-administered participant (section “3.1 Participant Flow and Sample Characteristics”), so the self-administered trajectories that carry the study’s positive findings all begin from a genuine pre-treatment baseline. Participation duration was itself participant-elected, and contributing participants fell from 18 at baseline to 3 by Visit 8; dropout may therefore be informative, since differential retention of participants who perceived benefit could manufacture apparent improvement across visits, and the missing-at-random assumption of the mixed models cannot be verified. The decline in contributing participants across the visit axis is largely structural: enrollment was rolling against a fixed close-out, so the number of monthly assessments available to a participant was determined mainly by enrollment date, and only 5 of the 26 participants who initiated treatment enrolled early enough to be eligible for a seventh or eighth assessment. Where a reason for ceasing participation was recorded, it related to burden, logistics, or intercurrent illness rather than to perceived lack of benefit, and no participant cited absence of benefit as a reason for stopping. Neither observation excludes informative dropout, since most participants who stopped completing assessments did so without comment, but neither provides positive evidence for it.

The study also combined two markedly different populations, self-administering participants and caregiver-administered care recipients, who differed substantially in age, education, and baseline impairment; although all efficacy and symptom analyses were conducted and reported separately by group, the small subgroup sizes preclude formal between-group comparison, and no conclusion drawn from one group should be generalized to the other or to a combined population. The sample was 96% European-identifying, with a mean of 16.0 years of education, so the findings should not be assumed to generalize to more diverse or less educated populations, in whom access, acceptability, adherence, and symptom reporting may all differ. Finally, verification of the model-proposed coding frame in the qualitative analysis was performed by a single unblinded author; independent double-coding was not feasible in this study, so the thematic analysis is best read as descriptive and hypothesis-generating.

Strengths include validated outcome measures administered at multiple timepoints, continuous adverse-event monitoring through multiple channels, mixed-effects modeling that handles missing data and variable observation counts appropriately, analysis sets defined prior to analysis, and a sensitivity analysis addressing the dose-duration confound.

### 4.9 Hypothesized mechanism: microtubule resonance

The mechanistic rationale that motivated this study is explicitly hypothetical, and we present it here as hypothesis rather than as established background. Microtubules, the cylindrical tubulin polymers that form the cytoskeletal infrastructure of neurons, have been theorized not only to provide structural support and regulate synapses but to encode memory (Craddock et al., 2012) and to support consciousness (Hameroff and Penrose, 2014; Hameroff, 2022). Microtubules oscillate and resonate at numerous frequencies, with self-similar dynamics spanning hertz to terahertz ranges, and when electromagnetic oscillating fields were applied to depolymerized tubulin the rate of assembly into microtubules peaked at a few megahertz (Sahu et al., 2014). Electromagnetic fields penetrate the skull poorly at these frequencies and, on reaching the brain, induce currents that tend to flow across the cortical surface rather than into the brain, whereas ultrasound operates noninvasively in the same megahertz range. Ultrasound acts directly on microtubule dynamics *in vitro*, slowing depolymerization (Gupta et al., 2013) and rescuing microtubule function from paclitaxel-induced cytotoxicity (Amaya et al., 2021).

These microtubule-based accounts remain hypothetical. Direct evidence that ultrasound at the intensities used here alters microtubule dynamics in the intact human brain is lacking, none of the studies cited demonstrate the mechanism transcranially *in vivo*, and the resonance and Orchestrated Objective Reduction framework on which it draws is contested. Ultrasound may equally act through mechanisms unrelated to microtubules, including activation of membrane mechanosensitive ion channels (Yoo et al., 2022) and enhanced clearance of interstitial and cerebrospinal fluid (Yoo et al., 2023). The present study was not designed to distinguish among these accounts and provides no evidence bearing on any of them. The rationale is advanced as a motivating hypothesis for the controlled animal and *in vitro* work proposed in section “4.7 Implications and Future Directions” rather than as an established mechanism.

### 4.10 Conclusion

This pilot study establishes that home-based, self-administered transcranial ultrasound with the US Pro2000 is feasible and well tolerated in adults with memory and cognitive difficulties, with only mild, transient adverse events during sustained real-world use. The efficacy picture is more tempered. Participants who administered and rated the intervention themselves reported reductions in depressive and cognitive symptoms over time, and these self-reports converged across monthly and daily measures and across quantitative and qualitative data. Yet the one objective cognitive measure did not change, and caregiver-rated recipients did not improve, so the signal cannot presently be separated from expectancy, natural fluctuation, or the uncontrolled design. These efficacy observations, moreover, pertain only to the younger, less-impaired, self-administered subgroup and should not be generalized to the older, more impaired caregiver-administered population, in whom no signal was observed. The appropriate conclusion is therefore a cautious one: TUS delivered in this accessible, low-burden format is safe and promising enough to warrant rigorous testing, but has not yet been demonstrated to be effective. Realizing its potential and clarifying whether the microtubule-based mechanisms hypothesized for ultrasound translate into cognitive benefit will require an adequately powered, double-blind, sham-controlled trial with objective and informant-based endpoints. Should such a trial confirm even a portion of the improvements reported here, a safe, non-invasive, home-deliverable intervention would represent a meaningful addition to a field with few effective options.

## Data Availability

The raw data supporting the conclusions of this article will be made available by the authors, without undue reservation.
