# Supplementary material for: Home-administered unfocused transcranial ultrasound in subjective cognitive decline and dementia: an open-label, uncontrolled feasibility pilot study
Source: Front Aging Neurosci. 2026 Sep 28;18:1937897. doi: 10.3389/fnagi.2026.1937897 (PMC13655026; doi:10.3389/fnagi.2026.1937897)
Supplement: Supplementary file 1 [file Table_1.docx]

Supplementary Material

**Table S1.**

*Self-administered efficacy outcomes: descriptive statistics by visit and mixed-effects model estimates.*

| **Outcome** | **Visit** | **n** | **Mean (SD)** | **Time β (SE)** | **Time p** | **Time p_FDR** | **Age β (SE)** | **Age p** | **Minutes β (SE)** | **Minutes p** |
| --- | --- | --- | --- | --- | --- | --- | --- | --- | --- | --- |
| **GAD-7 (anxiety, 0–21)** | 1 | 18 | 5.83 (3.81) | −0.197 (0.098) | 0.049 | 0.074 | 0.061 (0.076) | 0.441 | −0.0009 (0.0010) | 0.400 |
|  | 2 | 16 | 4.38 (3.48) |  |  |  |  |  |  |  |
|  | 3 | 12 | 4.00 (3.86) |  |  |  |  |  |  |  |
|  | 4 | 8 | 4.75 (2.66) |  |  |  |  |  |  |  |
|  | 5 | 7 | 4.29 (2.81) |  |  |  |  |  |  |  |
|  | 6 | 5 | 4.40 (1.95) |  |  |  |  |  |  |  |
|  | 7 | 4 | 4.00 (2.71) |  |  |  |  |  |  |  |
|  | 8 | 3 | 2.00 (2.00) |  |  |  |  |  |  |  |
| **CESD-5 (depression, 0–60)** | 1 | 18 | 20.00 (12.72) | −0.909 (0.309) | 0.005 | 0.015 | −0.232 (0.256) | 0.378 | −0.0020 (0.0035) | 0.566 |
|  | 2 | 16 | 14.50 (9.34) |  |  |  |  |  |  |  |
|  | 3 | 12 | 14.33 (10.85) |  |  |  |  |  |  |  |
|  | 4 | 8 | 15.50 (11.60) |  |  |  |  |  |  |  |
|  | 5 | 7 | 14.29 (13.24) |  |  |  |  |  |  |  |
|  | 6 | 5 | 11.20 (10.35) |  |  |  |  |  |  |  |
|  | 7 | 4 | 13.00 (13.22) |  |  |  |  |  |  |  |
|  | 8 | 3 | 8.00 (13.86) |  |  |  |  |  |  |  |
| **Inoue Total (cognition, 0–15)** | 1 | 17 | 14.24 (1.03) | −0.162 (0.100) | 0.113 | 0.113 | 0.020 (0.022) | 0.382 | −0.00001 (0.00033) | 0.972 |
|  | 2 | 14 | 13.79 (0.97) |  |  |  |  |  |  |  |
|  | 3 | 11 | 13.82 (0.87) |  |  |  |  |  |  |  |
|  | 4 | 8 | 13.75 (0.71) |  |  |  |  |  |  |  |
|  | 5 | 7 | 14.14 (1.07) |  |  |  |  |  |  |  |
|  | 6 | 5 | 13.80 (0.84) |  |  |  |  |  |  |  |
|  | 7 | 4 | 11.00 (5.35) |  |  |  |  |  |  |  |
|  | 8 | 3 | 14.33 (0.58) |  |  |  |  |  |  |  |

**Table S2**

*Caregiver-Administered Outcomes (MDS 3.0 Sections C and D): Descriptive Statistics by Visit and Mixed-Effects Model Estimates*

| **Outcome** | **Visit** | **n** | **Mean (SD)** | **Time β (SE)** | **Time p** | **Time p_FDR** | **Age β (SE)** | **Age p** | **Minutes β (SE)** | **Minutes p** |
| --- | --- | --- | --- | --- | --- | --- | --- | --- | --- | --- |
| C7 Short-term memory (0–1) | 1 | 8 | 0.25 (0.46) | −0.012 (0.018) | 0.497 | 0.621 | 0.024 (0.033) | 0.504 | 0.00005 (0.00025) | 0.862 |
|  | 2 | 7 | 0.29 (0.49) |  |  |  |  |  |  |  |
|  | 3 | 7 | 0.43 (0.53) |  |  |  |  |  |  |  |
|  | 4 | 6 | 0.17 (0.41) |  |  |  |  |  |  |  |
|  | 5 | 4 | 0.25 (0.50) |  |  |  |  |  |  |  |
|  | 6 | 4 | 0.25 (0.50) |  |  |  |  |  |  |  |
|  | 7 | 3 | 0.33 (0.58) |  |  |  |  |  |  |  |
|  | 8 | 2 | 0.00 (0.00) |  |  |  |  |  |  |  |
|  | 9 | 1 | 0.00 (—) |  |  |  |  |  |  |  |
| C8 Long-term memory (0–1) | 1 | 7 | 0.86 (0.38) | −0.003 (0.018) | 0.887 | 0.887 | 0.008 (0.038) | 0.843 | 0.00012 (0.00029) | 0.699 |
|  | 2 | 7 | 0.57 (0.53) |  |  |  |  |  |  |  |
|  | 3 | 7 | 0.43 (0.53) |  |  |  |  |  |  |  |
|  | 4 | 6 | 0.67 (0.52) |  |  |  |  |  |  |  |
|  | 5 | 4 | 0.75 (0.50) |  |  |  |  |  |  |  |
|  | 6 | 4 | 0.75 (0.50) |  |  |  |  |  |  |  |
|  | 7 | 3 | 1.00 (0.00) |  |  |  |  |  |  |  |
|  | 8 | 2 | 1.00 (0.00) |  |  |  |  |  |  |  |
|  | 9 | 1 | 1.00 (—) |  |  |  |  |  |  |  |
| C9 Memory/recall (0–4) | 1 | 8 | 1.50 (0.93) | 0.065 (0.047) | 0.175 | 0.438 | 0.076 (0.092) | 0.451 | 0.00012 (0.00071) | 0.871 |
|  | 2 | 7 | 1.71 (1.38) |  |  |  |  |  |  |  |
|  | 3 | 7 | 2.00 (1.53) |  |  |  |  |  |  |  |
|  | 4 | 6 | 2.17 (1.17) |  |  |  |  |  |  |  |
|  | 5 | 4 | 2.00 (1.41) |  |  |  |  |  |  |  |
|  | 6 | 4 | 1.75 (1.50) |  |  |  |  |  |  |  |
|  | 7 | 3 | 2.33 (1.53) |  |  |  |  |  |  |  |
|  | 8 | 2 | 2.50 (0.71) |  |  |  |  |  |  |  |
|  | 9 | 1 | 1.00 (—) |  |  |  |  |  |  |  |
| C10 Decision-making (0–3) | 1 | 8 | 1.63 (1.19) | 0.053 (0.029) | 0.076 | 0.380 | 0.027 (0.070) | 0.713 | −0.00061 (0.00054) | 0.307 |
|  | 2 | 7 | 1.57 (1.13) |  |  |  |  |  |  |  |
|  | 3 | 7 | 1.71 (1.11) |  |  |  |  |  |  |  |
|  | 4 | 6 | 1.50 (1.05) |  |  |  |  |  |  |  |
|  | 5 | 4 | 1.75 (0.96) |  |  |  |  |  |  |  |
|  | 6 | 4 | 1.75 (0.50) |  |  |  |  |  |  |  |
|  | 7 | 3 | 1.33 (0.58) |  |  |  |  |  |  |  |
|  | 8 | 2 | 1.50 (0.71) |  |  |  |  |  |  |  |
|  | 9 | 1 | 1.00 (—) |  |  |  |  |  |  |  |
| D1 Mood, PHQ-9 (0–27) | 1 | 8 | 6.63 (4.57) | 0.255 (0.244) | 0.304 | 0.507 | −0.127 (0.368) | 0.745 | −0.00299 (0.00283) | 0.340 |
|  | 2 | 7 | 6.71 (6.58) |  |  |  |  |  |  |  |
|  | 3 | 7 | 6.86 (5.11) |  |  |  |  |  |  |  |
|  | 4 | 6 | 6.83 (6.34) |  |  |  |  |  |  |  |
|  | 5 | 4 | 6.50 (7.72) |  |  |  |  |  |  |  |
|  | 6 | 4 | 7.50 (7.59) |  |  |  |  |  |  |  |
|  | 7 | 3 | 6.00 (6.56) |  |  |  |  |  |  |  |
|  | 8 | 2 | 0.50 (0.71) |  |  |  |  |  |  |  |
|  | 9 | 1 | 2.00 (—) |  |  |  |  |  |  |  |

*Note.* Caregiver-administered analysis set (N = 8). Visit 1 = baseline; subsequent visits were scheduled at monthly intervals. n = participants contributing at each visit. Estimates are from linear mixed-effects models with random participant intercepts and Satterthwaite denominator degrees of freedom (fixed effects: visit, mean-centered age, mean-centered total device-use minutes); β (SE) and p are shown once per outcome on the baseline row. Minutes coefficients are per minute of cumulative device use. Time p_FDR reflects Benjamini–Hochberg correction across the five time (visit) effects; age and minutes p values are unadjusted. For C7, C8, C10, and D1, higher scores indicate greater impairment or mood severity; for C9, higher scores indicate better recall. Binary (C7, C8) and ordinal (C9, C10) outcomes were modeled with linear mixed-effects models for stability at this sample size, and their estimates are approximate. A pre-specified GEE sensitivity analysis confirmed the null time effects; its robust standard errors are unreliable at eight clusters and are not used for covariate inference.

**Table S3**

*Monthly Self-Report Symptom Outcomes by Group: Per-Visit Means and Mixed-Effects Model Estimates*

| **Outcome** | **1** | **2** | **3** | **4** | **5** | **6** | **7** | **8** | **9** | **Visit β (SE)** | **p** | **p_FDR** |
| --- | --- | --- | --- | --- | --- | --- | --- | --- | --- | --- | --- | --- |
| ***Self-administered group (n = 16)*** | | | | | | | | | | | | |
| Memory loss | 56.1 | 39.7 | 33.7 | 26.6 | 29.0 | 24.6 | 22.5 | 21.7 |  | −3.79 (1.28) | 0.005 | 0.019 |
| Word-finding | 43.1 | 40.3 | 35.4 | 37.9 | 24.7 | 25.6 | 19.8 | 36.7 |  | −2.91 (1.10) | 0.011 | 0.022 |
| Confusion | 25.5 | 17.6 | 12.6 | 11.5 | 15.0 | 11.2 | 5.0 | 11.7 |  | −2.11 (0.92) | 0.025 | 0.040 |
| Problem-solving | 43.6 | 28.8 | 28.6 | 24.8 | 22.1 | 16.2 | 21.5 | 21.3 |  | −3.16 (1.13) | 0.007 | 0.019 |
| Mood/behavior | 37.4 | 28.8 | 26.4 | 29.9 | 30.0 | 31.0 | 17.5 | 21.3 |  | −1.18 (1.09) | 0.285 | 0.285 |
| Impaired judgment | 27.1 | 24.2 | 16.2 | 16.6 | 22.6 | 15.2 | 9.0 | 23.7 |  | −1.80 (0.99) | 0.073 | 0.097 |
| Social withdrawal | 33.6 | 31.6 | 22.6 | 31.6 | 29.9 | 29.6 | 25.0 | 19.3 |  | −1.49 (1.11) | 0.188 | 0.215 |
| Composite (CI_Total) | 38.0 | 30.1 | 25.1 | 25.6 | 24.8 | 21.9 | 17.2 | 22.2 |  | −2.35 (0.81) | 0.005 | 0.019 |
| ***Caregiver-administered group (n = 7)*** | | | | | | | | | | | | |
| Memory loss | 74.9 | 74.1 | 66.1 | 68.7 | 70.8 | 82.5 | 79.3 | 80.0 | 40.0 | 1.25 (0.99) | 0.217 | 0.840 |
| Word-finding | 71.1 | 60.4 | 59.6 | 59.0 | 64.5 | 66.0 | 53.3 | 53.0 | 50.0 | 0.29 (0.97) | 0.764 | 0.873 |
| Confusion | 56.1 | 51.3 | 44.6 | 52.3 | 39.0 | 57.5 | 48.7 | 53.5 | 6.0 | −1.13 (1.14) | 0.329 | 0.840 |
| Problem-solving | 80.4 | 60.9 | 67.9 | 64.3 | 67.2 | 73.2 | 65.3 | 74.0 | 65.0 | 0.87 (1.06) | 0.420 | 0.840 |
| Mood/behavior | 31.0 | 26.0 | 26.1 | 27.3 | 12.2 | 19.5 | 25.0 | 22.5 | 5.0 | −1.92 (1.17) | 0.110 | 0.840 |
| Impaired judgment | 67.1 | 58.6 | 54.0 | 62.7 | 56.8 | 60.0 | 33.3 | 75.0 | 25.0 | −0.14 (1.05) | 0.896 | 0.896 |
| Social withdrawal | 46.3 | 49.3 | 37.9 | 43.8 | 47.2 | 43.2 | 22.0 | 15.0 | 20.0 | −0.56 (1.25) | 0.657 | 0.873 |
| Composite (CI_Total) | 61.0 | 54.4 | 50.9 | 54.0 | 51.1 | 57.4 | 46.7 | 53.3 | 30.1 | −0.22 (0.62) | 0.724 | 0.873 |

*Note.* Values under columns 1–9 are observed means of the 0–100 symptom sliders at each visit (visit 1 = baseline; higher scores indicate greater symptom burden). Estimates are from linear mixed-effects models with random participant intercepts and Satterthwaite denominator degrees of freedom (fixed effects: visit, mean-centered age, mean-centered total device-use minutes). Visit β (SE) is the per-visit change; a negative value indicates symptom reduction. p_FDR reflects Benjamini–Hochberg correction across the eight outcomes within each group. Self-administered n declined from 16 at baseline to 3 by visit 8; caregiver-administered n declined from 7 to 1 by visit 9. Age and device-use minutes were not significantly associated with any outcome in either group (all p ≥ .26).

**Table S4**

*Themes from open-ended daily symptom-change comments*

| **Theme** | **Ppts, n** | **Representative comment** |
| --- | --- | --- |
| **Positive-change comments** |  |  |
| Memory and recall | 9 | *“short term memory seems a little better… less at a loss for the right word”* |
| Mental clarity, focus, sharpness | 8 | *“feeling sharper… a clearer head in general”* |
| Mood and general well-being | 8 | *“slight mood improvement… more upbeat”* |
| Calm / reduced anxiety | 8 | *“I am more calm which allows [me] to think with clarity”* |
| Engagement in activities and hobbies | 8 | *“read a book for two hours… [had] not done [so]… for the past two years”* |
| Energy / reduced daytime sleepiness | 5 | *“greater sense of well being and more energy”* |
| Social engagement and communication | 4 | *“asked people appropriate questions… a first in years”* |
| Sleep quality | 4 | *“I am sleeping better”* |
| Dream recall / vividness | 3 | *“remembering longer portions of dreams upon waking”* |
| Reduced involuntary movements / restlessness | 2 | *“involuntary muscular movements seem diminished during treatment”* |
| **Negative-change comments** |  |  |
| Cognitive fluctuation/worsening (fog, confusion, memory) | 6 | *“increasing memory and cognition issues”* |
| Headache | 4 | *“headache — not too bad”* |
| Fatigue / sleepiness | 4 | *“I feel more fatigued at night th[a]n usual”* |
| Mood / irritability | 4 | *“more cranky, frustrated with outer circumstances”* |
| Visual / eye strain | 2 | *“eyes feel tired or strained but not painful”* |
| Sleep disturbance | 2 | *“sleep has been disrupted… difficult getting to sleep”* |
| Neck discomfort | 1 | *“some neck discomfort”* |
| Tremor / edginess | 1 | *“edgy and shaky… don’t know that the ultrasound is why”* |

*Note.* Themes derived from free-text responses to the positive- and negative-change items of the Daily Assessment Form (294 positive comments from 21 participants; 60 negative comments from 14 participants) via AI-assisted extraction with manual review. Counts are the number of participants contributing at least one comment to a theme; themes are not mutually exclusive and counts are not additive. Mention frequency is not reported because it was skewed by differential reporting volume across participants.

Representative comments are de-identified and lightly edited for spelling and clarity; bracketed text indicates editorial insertions.
